# The Success of *Acinetobacter* Species; Genetic, Metabolic and Virulence Attributes

**DOI:** 10.1371/journal.pone.0046984

**Published:** 2012-10-29

**Authors:** Anton Y. Peleg, Anna de Breij, Mark D. Adams, Gustavo M. Cerqueira, Stefano Mocali, Marco Galardini, Peter H. Nibbering, Ashlee M. Earl, Doyle V. Ward, David L. Paterson, Harald Seifert, Lenie Dijkshoorn

**Affiliations:** 1 Department of Microbiology, Monash University, Melbourne, Victoria, Australia; 2 Department of Infectious Diseases, Alfred Hospital, Melbourne, Victoria, Australia; 3 Division of Infectious Diseases, Beth Israel Deaconess Medical Center, Boston, Massachusetts, United States of America; 4 Department of Infectious Diseases, Leiden University Medical Center, Leiden, The Netherlands; 5 Department of Genetics and Center for Proteomics and Bioinformatics, Case Western Reserve University, Cleveland, Ohio, United States of America; 6 Agrobiology and Pedology Centre, Agricultural Research Council (CRA-ABP), Florence, Italy; 7 Department of Evolutionary Biology, University of Florence, Florence, Italy; 8 Broad Institute, Cambridge, Massachusetts, United States of America; 9 University of Queensland Centre for Clinical Research, Royal Brisbane and Women's Hospital Campus, Brisbane, Queensland, Australia; 10 Institute for Medical Microbiology, Immunology, and Hygiene, University of Cologne, Cologne, Germany; University of Florida, United States of America

## Abstract

An understanding of why certain *Acinetobacter* species are more successful in causing nosocomial infections, transmission and epidemic spread in healthcare institutions compared with other species is lacking. We used genomic, phenotypic and virulence studies to identify differences between *Acinetobacter* species. Fourteen strains representing nine species were examined. Genomic analysis of six strains showed that the *A. baumannii* core genome contains many genes important for diverse metabolism and survival in the host. Most of the *A. baumannii* core genes were also present in one or more of the less clinically successful species. In contrast, when the accessory genome of an individual *A. baumannii* strain was compared to a strain of a less successful species (*A. calcoaceticus* RUH2202), many operons with putative virulence function were found to be present only in the *A. baumannii* strain, including the *csu* operon, the acinetobactin chromosomal cluster, and bacterial defence mechanisms. Phenotype microarray analysis showed that compared to *A. calcoaceticus* (RUH2202), *A. baumannii* ATCC 19606^T^ was able to utilise nitrogen sources more effectively and was more tolerant to pH, osmotic and antimicrobial stress. Virulence differences were also observed, with *A. baumannii* ATCC 19606^T^, *A. pittii* SH024, and *A. nosocomialis* RUH2624 persisting and forming larger biofilms on human skin than *A. calcoaceticus*. *A. baumannii* ATCC 19606^T^ and *A. pittii* SH024 were also able to survive in a murine thigh infection model, whereas the other two species were eradicated. The current study provides important insights into the elucidation of differences in clinical relevance among *Acinetobacter* species.

## Introduction

In contemporary medicine, certain *Acinetobacter* species have proven to be highly successful in their ability to cause outbreaks and develop antibiotic resistance [1], [2]. However, great diversity exists in the clinical importance of the various species, with some being dominant as human pathogens and others merely acting as colonizing or environmental organisms [2]. To date, with the recent description of the novel species *Acinetobacter pittii* (former name *Acinetobacter* genomic species [gen. sp.] 3) and *Acinetobacter nosocomialis* (former name *Acinetobacter* gen. sp. 13TU) [3], the genus *Acinetobacter* comprises 27 validly named species and 9 DNA–DNA hybridization groups (gen. sp.) with provisional designations. *A. baumannii* has long been considered the most clinically important species, with the greatest number of healthcare–related outbreaks and reports of multidrug resistance. More recently, and likely as a consequence of improved laboratory identification, *A. pittii* and *A. nosocomialis* have also surfaced as clinically significant, with increasing reports of outbreaks and antibiotic resistance [4], [5], [6], [7], [8], [9]. Species that have less commonly been associated with human disease include *A. lwoffii*, *A. junii*, and *A. haemolyticus*, and some species have only been identified as colonizing human skin or very rarely described as causing human disease, such as *A. johnsonii* and *A. radioresistens* [2], [6]. To our knowledge, *A. calcoaceticus* has never been implicated in serious human disease [2]. However, given the difficulty in phenotypically differentiating it from *A. baumannii*, *A. pittii* and *A. nosocomialis*, these species are often grouped together in diagnostic microbiology laboratories as the ‘*A. calcoaceticus* – *A. baumannii* complex’.

Thus far, the attributes that make one *Acinetobacter* species more adept at causing human outbreaks and disease than another are poorly understood. Previous studies have shown that *A. baumannii* has the ability to survive in both wet and dry conditions in the hospital environment [10], [11], [12]. A recent clinical study showed that relative to *A. nosocomialis*, *A. baumannii* was an independent predictor of mortality [4]. A variety of virulence mechanisms have been identified in *A. baumannii*, including siderophore–mediated iron acquisition systems, biofilm formation, adherence and outer membrane protein function, the lipopolysaccharide (LPS), capsule formation, and quorum–sensing [13], [14], [15], [16], [17], [18], [19], [20]. Significantly less is known about the non–*baumannii* species. In this study, we used genomics, phenotype microarray analyses and virulence studies, to identify species characteristics that may explain why some *Acinetobacter* species are successful as human pathogens and others are not.

## Results

### Genome Characteristics of the *Acinetobacter* Species

As shown in Table 1, 14 genomes were included in this analysis, covering nine different *Acinetobacter* species (species names will be used for non–*baumannii* species throughout). Eight strains were sequenced as part of this study with mean coverage of 22–fold. Overall, the species that make up the *A. calcoaceticus* – *A. baumannii* complex had the largest genomes, with *A. radioresistens* having the smallest (3.16 Mb). Genome sizes of strains within the *A. baumannii* species varied by up to 289 Kb. The number of genes corresponded to genome size, ranging from 3,690 in *A. baumannii* to 2,874 in *A. radioresistens* (Table 1). Phylogenetic analysis showed that the species that make up the *A. calcoaceticus* – *A. baumannii* complex were most closely related (Figure S1). The other species formed distinct phylogenetic branches.

**Table 1:** Characteristics of the bacterial strains used in this study.

Bacterial species^1^	Strain name	Origin (Place, year)	Source	Genome Size (Mb)	No. of Genes	Reference for Genome Sequence	Genbank Accession No.
*A. baumannii* 2	ATCC 19606^T^	Unknown, before 1949	Urine	3.97	3,766	This study	ACQB00000000
*A. baumannii*	ATCC 17978	Unknown, ∼1951	Unknown	3.98	3,791	[50]	CP000521
*A. baumannii*	AB0057	Washington, D.C., USA, 2003–2005	Blood	4.05	3,853	[51]	CP001182
*A. baumannii*	AB307–0294	Buffalo, NY, USA, 1994	Blood	3.76	3,458	[51]	CP001172
*A. baumannii*	AYE	Le Cremlin–Bicêtre, FR, 2001	Urine	3.94	3,607	[52]	CU459141
*A. baumannii*	ACICU	Rome, IT, 2005	CSF	3.90	3,667	[53]	CP000863
*A. calcoaceticus* 2	RUH2202	Malmoe, SE, 1980–82	Wound	3.88	3,566	This study	ACPK00000000
*A. pittii* 2	SH024	Cologne, DE, 1993	Skin (axilla)	3.97	3,689	This study	ADCH00000000
*A. nosocomialis* 2	RUH2624	Rotterdam, NL, 1987	Skin (forehead)	3.87	3,631	This study	ACQF00000000
*A. lwoffii*	SH145	Cologne, DE, 1994	Skin (hand)	3.48	3,134	This study	ACPN00000000
*A. junii*	SH205	Cologne, DE, 1994	Skin (perineum)	3.46	3,186	This study	ACPM00000000
*A. radioresistens*	SH164	Cologne, DE, 1994	Skin (forehead)	3.16	2,874	This study	ACPO00000000
*A. johnsonii*	SH046	Cologne, DE, 1994	Skin (perineum)	3.69	3,363	This study	ACPL00000000
*A. baylyi*	ADP1	Atlanta, GA, USA, before 1958	Soil	3.60	3,325	[54]	CR543861

DE, Germany; FR, France; IT, Italy; NL, The Netherlands; SE, Sweden; US, United States.

1 *A. baumannii* SDF was not included in this study due to its significantly reduced genome size and gene number compared to the *A. baumannii* isolates derived from human sources.

2 Representative strains of the *A. calcoaceticus* – *A. baumannii* complex that were analysed in detail.

### Analysis of the *A. baumannii* Core Genome

**Figure 1:**
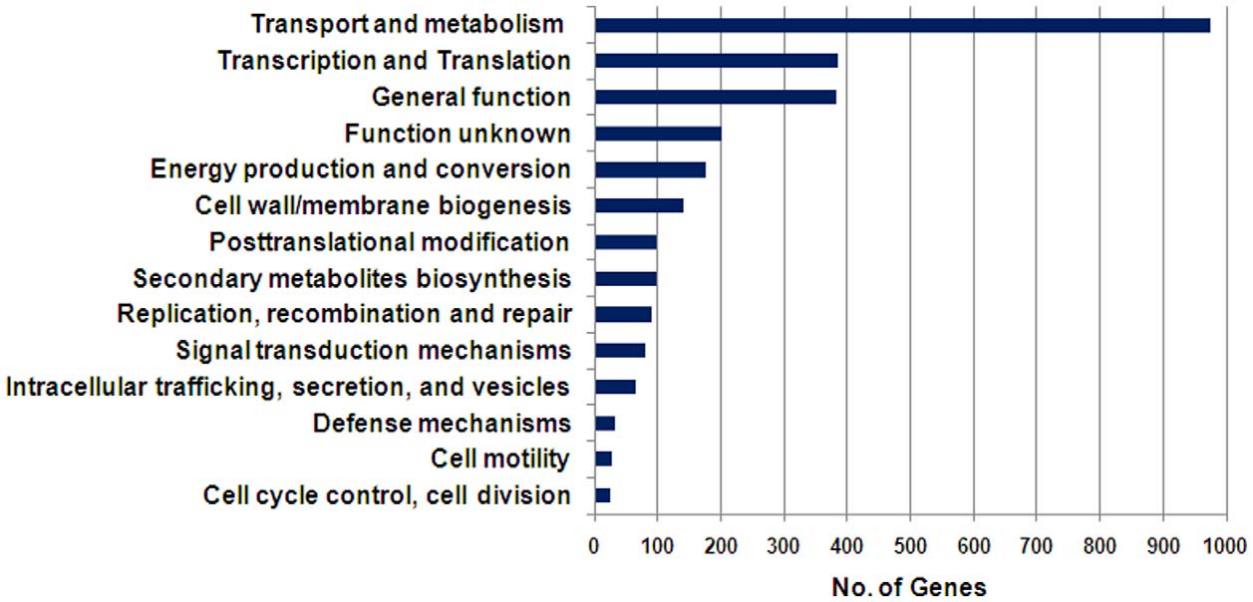
*A. baumannii* core genome. Functional distribution of the genes found in all six *A. baumannii* strains included in this study.

To understand the genetic core of *A. baumannii*, we first analysed the orthologous genes found in all six *A. baumannii* genomes. This analysis yielded 2,800 genes, indicating that the accessory genome, defined as the genes not found within the core *A. baumannii* genome, varied between 658–1,053 genes depending on the strain. A distribution of the *A. baumannii* core genome based on functional gene categories is shown in Figure 1. Apart from genes of general or unknown function, genes related to molecule transport and metabolism were most abundant (35%), including amino acid (11%), carbohydrate (5%), lipid (5%), nucleotide (3%), coenzyme (4%) and inorganic ion (7%) processing. Interestingly, despite *Acinetobacter* deriving its name from akineto meaning non–motile, *A. baumannii* has several core cell motility genes. These include a type IV pilus apparatus and pilus assembly genes (*pilB*, *pilW*, *pilL*, *pilJ*, *pilI*, *pilY1*, *pilQ*, *pilO*, *pilN*, *pilM*), fimbrial biogenesis genes (*fimT*, *pilZ*), and twitching motility genes (*pilU*, *pilT*, which are important for pilus retraction). In fact, it has recently been shown that *A. baumannii* is motile under certain conditions [21], [22], and this may play an important role in its ability to colonize and spread on surfaces, and to form biofilms [23].

### Comparison of *A. baumannii* Genome with Other *Acinetobacter* Species

To begin to decipher the genetic attributes that may help explain why some *Acinetobacter* species are clinically more significant than others, we sought to identify genes only present in pathogenic species of *Acinetobacter* (the six *A. baumannii* genomes, and the *A. pittii* and *A. nosocomialis* genome) and not present in the other species. This analysis identified 51 genes, including 12 putative operons, shared among these eight genomes that were not present in the other species (Table S1). Importantly, one of these operons was the *csu* operon, which includes six genes, and codes for proteins involved in a chaperone – usher pili assembly system [17]. This operon appears important for pili assembly, adherence to abiotic surfaces and biofilm formation [17]. The finding that this operon is only present in pathogenic species of *Acinetobacter* further highlights its potential role in determining the clinical success of these species. The predominant functional categorization of the remaining genes was in molecule transport and metabolism, and transcription (Table S1).

### Comparison of Specific Strains of the *A. calcoaceticus* – *A. baumannii* Complex

**Figure 2:**
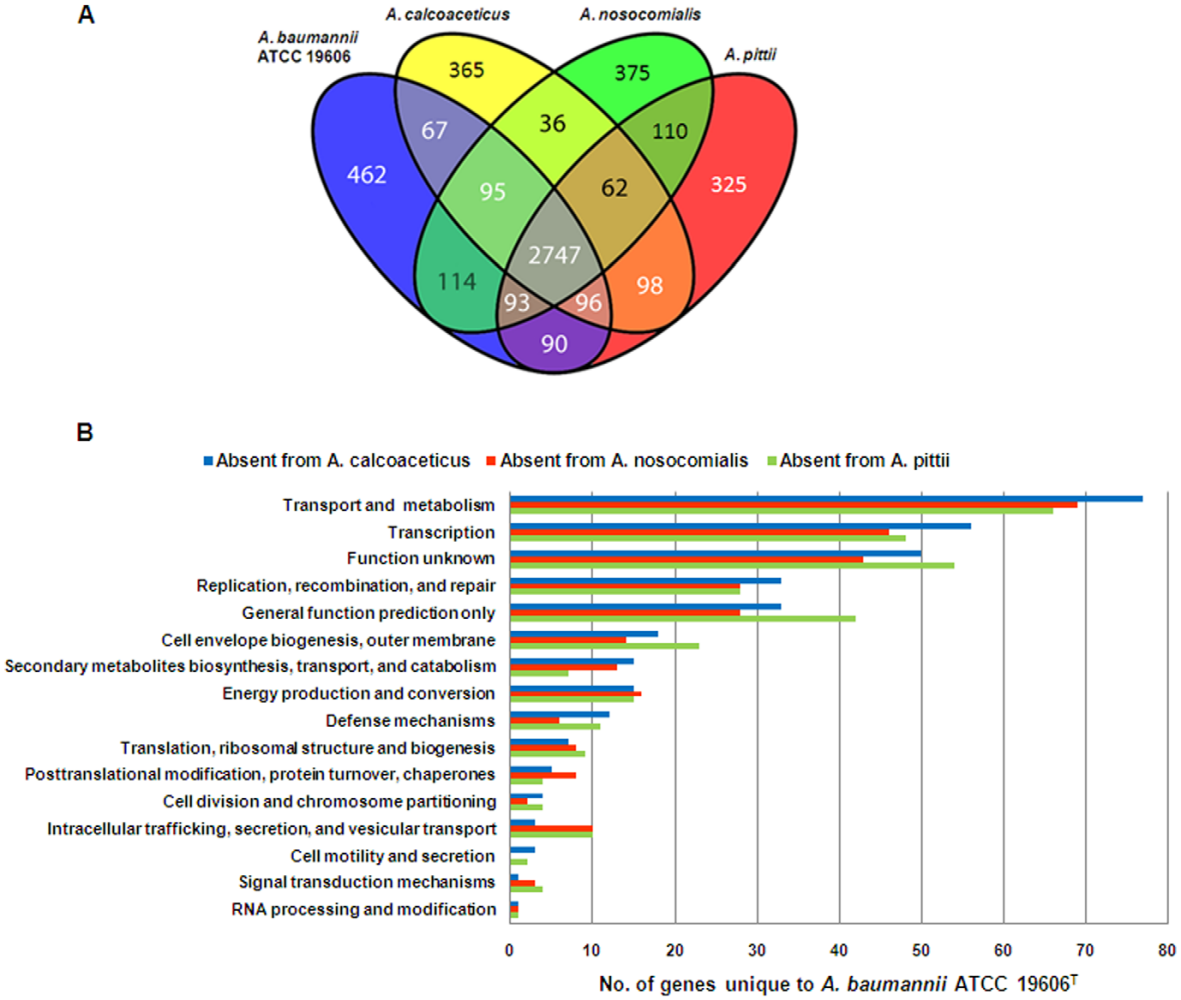
Distribution of genes in individual strains of the *A. calcoaceticus – A. baumannii* complex. (**A**) Venn diagram showing the number of overlapping genes between the four strains that make up the *A. calcoaceticus* – *A. baumannii* complex. (**B**) Pairwise comparisons of the number of genes present in *A. baumannii* ATCC 19606^T^ but absent in each of *A. calcoaceticus* (blue), *A. pittii* (green) and *A. nosocomialis* (red).

The data presented thus far provide some evidence that a small number of core genes may partly explain the clinical success of certain *Acinetobacter* species; however the number of genes that differed between pathogenic and less pathogenic species was few, suggesting that additional genetic characteristics that distinguish these strains may be found among the accessory genomes. To interrogate the accessory genome in more detail, we analysed representative strains from the four species that make up the *A. calcoaceticus* – *A. baumannii* complex (Table 1). A distribution of genes is shown in Figure 2A. A total of 2747 genes were common to all four species, with the greatest number of species-specific genes observed in *A. baumannii* ATCC 19606^T^ (Figure 2A). Based on clusters of orthologous group (COG) functional classification, the distribution of genes unique to *A. baumannii* ATCC 19606^T^ compared to each one of the other three strains (individual pairwise comparisons) was similar, with the greatest number of ATCC 19606^T^-specific genes having a presumptive role in amino acid, carbohydrate and lipid transport and metabolism, and transcription (Figure 2B).

**Figure 3:**
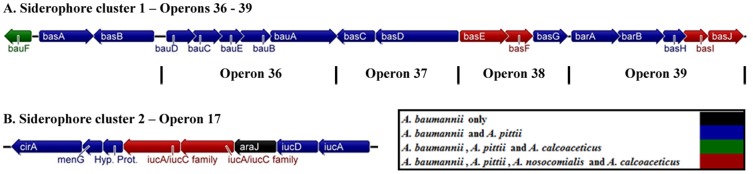
Genetic organisation and conservation of the siderophore clusters found in *A. baumannii* ATCC 19606^T^ and not in *A. calcoaceticus*. (**A**) Siderophore cluster 1 (operons 36–39) is known as the acinetobactin chromosomal cluster, and (**B**) siderophore cluster 2 (operon 17) (See Table 2 for details about the operons). The presence of homologues for each gene in *A. pittii*, *A. nosocomialis*, and *A. calcoaceticus* is shown.

Of most interest was the comparison between *A. baumannii* ATCC 19606^T^ and *A. calcoaceticus*. This comparison identified 759 genes present in *A. baumannii* ATCC 19606^T^ and not in *A. calcoaceticus* (Table S2). Of these, only 169 were found in the other five *A. baumannii* genomes analysed in this study, indicating that the majority (78%) were part of the accessory genome of *A. baumannii* ATCC 19606^T^. Of the 759 genes, 333 had a COG classification, and they were significantly overrepresented in several functions necessary for basic bacterial growth and survival, including transcription (56 genes), DNA replication, recombination, and repair (33 genes), amino acid, inorganic ion and carbohydrate transport and metabolism (66 genes), and cell envelope biogenesis and outer membrane function (19 genes). Among the 333 genes with a COG classification, there were 69 putative operons (Table S2) that were enriched in virulence–related genes, including those involved in siderophore transport and biosynthesis, LPS biosynthesis, pili and biofilm formation, Curli fimbriae assembly, and bacterial phage resistance mechanisms (Table 2). Several operons responsible for iron handling were identified, including the acinetobactin chromosomal locus (operons 36–39, Table 2), encoding a key *Acinetobacter* siderophore [24], [25]. The genetic organisation of this locus and homologues in *A. pittii*, *A. nosocomialis* and *A. calcoaceticus* are shown in Figure 3A. *A. calcoaceticus* and *A. nosocomialis* lacked the full complement of genes that make this locus (Table 2 and Figure 3A). We also identified a more recently described siderophore operon (operon 17, Table 2 and Figure 3B) [21], made up of eight genes, with *A. baumannii* ATCC 19606^T^ being the only strain with the full complement of genes, and *A. nosocomialis* and *A. calcoaceticus* being deficient in most of them.

**Table 2:** Select operons with putative virulence function found in *A. baumannii* ATCC 19606^T^ and not in *A. calcoaceticus*.

Operon ID	Function	Genes	*A. baumannii* ATCC 19606^T^ ORFs^1^	Homologues average similarity (ID% ± SD)^2^	
				*A. pittii*	*A. nosocomialis*
2	Pili assembly and biofilm form.	*csuAB/ABCDE*	ACIB1v1_50001–6	91.8±7.1	91.8±7.1
17	Siderophore transp. bios.	*cirA*, *menG*, *iucA/C, araJ*, *rhbE/C*	ACIB1v1_160094–101^3^	46.2±17.9	31.1±3.7
29	Phage resistance	*cas1, csy1, csy2, csy3, csy4*	ACIB1v1_260071–75	–	–
36	Siderophore transp. bios.	*bauD*, *bauC*, *bauE*, *bauB* and *bauA*	ACIB1v1_480066–70	97.8±1.0	–
37	Siderophore transp. bios.	*basC* and *basD*	ACIB1v1_480071–72	97.4±1.0	–
38	Siderophore transp. bios.	*basE, basF, basG*	ACIB1v1_480073–75	97.6±0.3	47.8±0.4
39	Siderophore transp. bios.	*barA, barB, basH, basI, basJ*	ACIB1v1_480076–80	95.1±6.3	32.8±0.8
40	Siderophore transp. bios.	*tonB*, PEPN	ACIB1v1_490004–5	97.0±0.3	97.5±0.3
46	Cell motility and secretion	*pilA*	ACIB1v1_560044–45	60.2±14.5	73.3±0.3
47	LPS biosynthesis	*lpsC* and *lpsE*	ACIB1v1_600015–16^4^	33.8^5^	68.2±29.8
50	Curli fimbriae assembly	*csgG*	ACIB1v1_700078–80	94.8±1.1	–
56	LPS biosynthesis	*wzx*, *degT*, *wbbJ*, *mviM* and *vipA*	ACIB1v1_740018–22^6^	42.6±23.8	48.3±17.2

Bios., Biosynthesis; Form., Formation; ID, Identity; SD, Standard Deviation; Transp., Transport.

1 Based on Microbial Genome Annotation Platform (www.cns.fr/agc/mage) [55].

2 Expressed as the average identity at the nucleotide level ± standard deviation.

3 Only three, two and two genes (out of eight) are found in *A. pittii*, *A. nosocomialis* and *A. calcoaceticus*, respectively. The homologues identified exhibited low similarity.

4 Both genes belong to an LPS operon that spans from ORF ACIB1v1_600009 to 16, and which is only partially present within *A. calcoaceticus* (three of eight genes are absent).

5 *lpsC* is absent from *A. pittii* genome.

6 Only *vipA* is present in *A. calcoaceticus* and exhibited moderate similarity. The operon is poorly conserved and partially present also in *A. pittii* and *A. nosocomialis*.

Several genes related to bacterial defence mechanisms were also observed only in *A. baumannii* ATCC 19606^T^ and not in *A. calcoaceticus*, including those coding for ABC transporters, and CRISPR – (Cas) and phage–resistance proteins (Table S2). Clustered regularly interspaced short palindromic repeats (CRISPRs) are recently described adaptive bacterial immune mechanisms that protect bacteria from invading foreign genetic elements such as bacteriophages [26], [27]. Such systems, when combined with other phage resistance mechanisms, may provide a survival benefit to the bacterial host [26]. The *A. baumannii* ATCC 19606^T^ CRISPR system includes *cas1* and *cas3*; however we could not locate *cas2*, which is thought to be required with *cas1* to form a functional CRISPR system [27]. The CRISPR operon was not found in the *A. calcoaceticus*, *A. nosocomialis* or *A. pittii* strains.

### Comparison of the Metabolic Versatility of Specific Strains of the *A. calcoaceticus* – *A. baumannii* Complex

**Figure 4:**
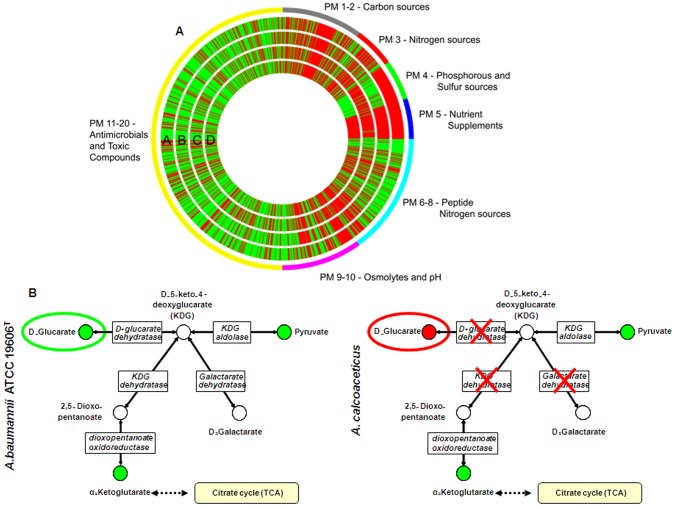
Metabolic diversity of specific strains of the *A. calcoaceticus* – *A. baumannii* complex. (**A**) Phenotype Microarray (PM) comparative results showing the number of compounds used (green) or not used (red) by *A. baumannii* ATCC 19606^T^ [A], *A. nosocomialis* [B], *A. pittii* [C] *and A. calcoaceticus* [D]. The external circle and PM number represent the Biolog plate number. (**B**) *A. baumannii* ATCC 19606^T^ is able to metabolise the carbon source, D–glucarate and produce α–Ketoglutarate through the functional enzymes, D–glucarate dehydrogenase and KDG dehydratase. α–Ketoglutarate is then utilized in the citrate cycle. These enzymes are not found in *A. calcoaceticus*.

Given the predominance of metabolism genes differentiating pathogenic and less pathogenic strains (Table S1 and S2), we analyzed the metabolic profile of the four species of the *A. calcoaceticus* – *A. baumannii* complex using phenotype microarrays. Of the 1920 conditions tested, the four species shared 1356 metabolic responses (70.6%), of which 795 compounds or conditions could be utilized by all the species and 561 by none of them. A summary of the entire metabolic profile of the four species is shown in Figure 4A. *A. baumannii* ATCC 19606^T^ appeared to utilize peptide nitrogen sources (PM 6–8) more effectively and to be more tolerant to pH stress (PM 10) than the other three species. *A. baumannii* ATCC 19606^T^ and *A. pittii* had a reduced ability to utilise most of the phosphorus and sulfur sources (PM 4) (Figure 4A).

We then focused on the most clinically disparate of the species, and compared *A. baumannii* ATCC 19606^T^ with *A. calcoaceticus* in more detail. In 195 conditions (Table S3), *A. baumannii* ATCC 19606^T^ was significantly more metabolically active than *A. calcoaceticus*. These conditions comprised 10 carbon sources, 105 nitrogen sources (of which 98 were di– and tri–peptides) and 80 stress conditions, of which 26 related to osmotic and pH stress and 54 related to the presence of antimicrobials and other cytotoxic compounds (Table S3). Apart from the likely survival advantage inferred by the greater ability of *A. baumannii* ATCC 19606^T^ to metabolise in the presence of osmotic, pH and antimicrobial exposure, one of the carbon sources utilized by this strain was D–glucarate. D–glucarate is found in the human body and has been shown to be a carbon source utilized by a range of gram–negative bacteria [28], [29]. D–glucarate catabolism generates α–ketoglutarate, which enhances the citric acid cycle (Figure 4B). Recently, over–expression of the citric acid cycle was shown to occur in an *A. baumannii* strain with increased virulence in the presence of ethanol [30].

### Virulence Differences between Strains of the *A. calcoaceticus* – *A. baumannii* Complex

**Figure 5:**
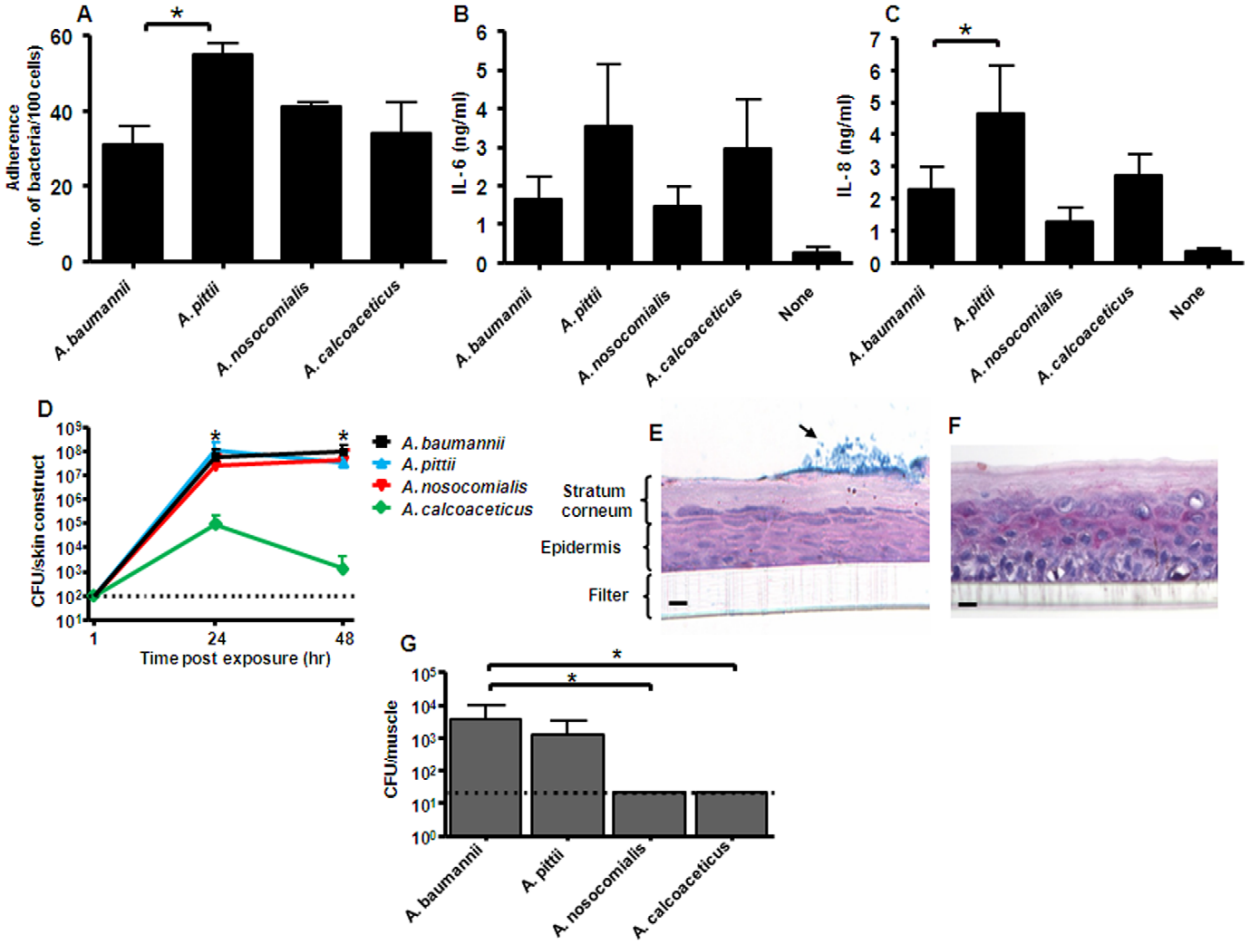
Virulence attributes of individual strains belonging to the *A. calcoaceticus – A. baumannii* complex. (**A**) Adherence of *A. baumannii* ATCC 19606^T^, *A. pittii*, *A. nosocomialis* and *A. calcoaceticus* to human bronchial epithelial cells after 1 hour. Results are expressed as mean number of bacteria per 100 epithelial cells ± standard deviation (SD) of two independent experiments performed in duplicate. The asterisk signifies statistical significance (*P*<0.05) between *A. pittii* and *A. baumannii*. The comparison between *A. pittii* and *A. calcoaceticus* was also significant (*P*<0.05). (**B**) Levels of IL–6 and (**C**) IL–8 in the culture medium of human bronchial epithelial cells after 24 hour stimulation with specific strains of the *A. calcoaceticus* – *A. baumannii* complex. Results are expressed as mean levels of IL–6 and IL–8 (in ng/ml) ± SD of three independent experiments. Asterisk signifies statistical significance (*P*<0.05) between *A. pittii* and *A. baumannii*. The comparison between *A. pittii* and *A. calcoaceticus* was also significant (*P*<0.05).. (**D**) Persistence and biofilm formation of *A. baumannii* ATCC 19606^T^ (squares), *A. pittii* (upward triangles), *A. nosocomialis* (downward triangles) and *A. calcoaceticus* (diamonds) on three–dimensional human skin constructs. Results are expressed as mean CFU per skin construct ± SD of three independent experiments. Dotted line represents the lower limit of detection. Asterisk signifies statistical significance comparing *A. calcoaceticus* with *A. baumannii* ATCC 19606^T^ (*P*<0.05) (**E**) Alcian–blue PAS staining shows biofilm formation (black arrow) on human skin constructs by *A. baumannii* ATCC 19606^T^ but not by (**F**) *A. calcoaceticus*. Scale bar is equivalent to 20 µm. (**G**) Approximately 1×10^4^ CFU were injected in the thigh muscles of neutropenic mice and the number of viable bacteria was determined after 48 hrs. Results are expressed as mean number of bacteria (in CFU/muscle) ± SD from three animals. Dotted line represents lower limit of detection. Asterisk signifies statistical significance (*P*<0.05) between *A. baumannii* and *A. nosocomialis* or *A. calcoaceticus*.

Given the differences in the number of putative operons with virulence function that were observed between the four species of the *A. calcoaceticus* – *A. baumannii* complex, we performed a range of *in vitro* and *in vivo* virulence studies to characterise further the functional significance of their genetic differences. Given the predilection of *A. baumannii* to colonise or infect the respiratory tract, we first analysed the interaction of the four species with human bronchial epithelial cells. All strains could adhere to human bronchial epithelial cells and induce a pro-inflammatory cytokine response (IL-6 and IL-8). Adherence and IL-8 induction were most pronounced with *A. pittii* (Figure 5, A, B and C). Of note, cell monolayers remained intact and the morphology of the cells was not affected by any of the strains (data not shown).

Given the likely importance of biofilm formation to the success of *Acinetobacter* in hospitals, we next tested the four species in a unique biofilm assay. Thus far, the correlation between biofilm formation on abiotic surfaces and clinical significance has been poor [31]. Therefore, we used a novel assay that may predict the ability of *Acinetobacter* to colonise and form a biofilm on human skin [32]. Using a three–dimensional human skin construct, we observed that *A. baumannii* ATCC 19606^T^, *A. pittii* and *A. nosocomialis* were able to multiply rapidly and persist on human skin, whereas *A. calcoaceticus* grew to a significantly lower density (Figure 5D). In addition, biofilms of the former three species were visible on the stratum corneum after PAS-Alcian blue staining, whereas no such bacterial structures were seen for *A. calcoaceticus* (Figure 5, E and F).

Finally, we assessed the survival of the four strains in a neutropenic mouse thigh muscle infection model, which is a soft-tissue model previously used for *Acinetobacter* infection [33]. *A. calcoaceticus* and *A. nosocomialis* were eradicated from the mouse thigh muscles, whereas bacteria of *A. baumannii* ATCC 19606^T^ and *A. pittii* could be detected in the muscles after 48 hours (Figure 5G).

## Discussion

This study provides a combined genomic, phenotypic and virulence assessment of a range of *Acinetobacter* species that have been variably associated with humans. From a genomic analysis of nine different *Acinetobacter* species, we identified a small number of genes unique to pathogenic species. The majority of these genes are predicted to be important for molecule transport and metabolism but also included the putative virulence *csu* operon. Investigating the accessory genome of individual strains of the four species of the *A. calcoaceticus* – *A. baumannii* complex, we found a range of putative operons with predicted functions related to host survival and virulence in *A. baumannii* ATCC 19606^T^ but not in *A. calcoaceticus*. *A. pittii* appeared most similar to *A. baumannii* ATCC 19606^T^, whereas *A. nosocomialis* lacked several of these important operons, particularly the full repertoire of genes of the acinetobactin chromosomal locus. Phenotype microarray studies supported the genomic analysis in that *A. baumannii* ATCC 19606^T^ was able to utilise more carbon and nitrogen sources, and was more tolerant to a range of cellular stresses than *A. calcoaceticus*. Moreover, the pathogenic species were able to multiply and form biofilms on human skin significantly more than *A. calcoaceticus*. Only *A. baumannii* and *A. pittii* were able to survive in a mammalian thigh infection model.

As a consequence of improved laboratory speciation, it is becoming apparent that non–*baumannii* species, particularly *A. nosocomialis* and *A. pittii*, are clinically significant human pathogens. For example, in a recent study from Norway, *A. nosocomialis* was the most common species (47%) isolated from blood cultures over a three–year period, followed by *A. pittii* (20%) [34]. With regard to their clinical impact, a more contemporary study has shown that relative to *A. nosocomialis*, bacteremia with *A. baumannii* was an independent predictor of mortality [4]. Interestingly, and consistent with our study findings, there was no significant difference between *A. baumannii* and *A. pittii*, however the number of patients in the *A. pittii* group was small [4]. Genetically and metabolically, we showed that *A. pittii* appeared similar to *A. baumannii*, and they also behaved similarly in the mammalian infection model. Despite its description of causing bloodstream infection, *A. nosocomialis* lacked several of the putative virulence related operons, particularly the acinetobactin siderophore cluster, and its phenotype was more closely aligned to *A. calcoaceticus*, both of which may explain its failure to survive in the murine model and its association with a lower mortality in clinical studies [4].

Within mammalian hosts, free iron is often a scarce resource and for pathogenic organisms to survive *in vivo* they often utilize a range of iron scavenging systems. Such systems have been analysed across different *A. baumannii* strains [21], [35] however this is the first analysis, to our knowledge, of such genes in non–*baumannii* species. In addition to the acinetobactin chromosomal locus, we observed another siderophore cluster and a putative iron uptake receptor in *A. baumannii* ATCC 19606^T^ that was not present in *A. calcoaceticus*. This second cluster (operon 17 in Table 2) is a recently described siderophore cluster made up of eight genes that is well conserved across *A. baumannii* strains [21]. The full repertoire of genes from this cluster was not found in *A. pittii*, *A. nosocomialis*, and *A. calcoaceticus*, and the few homologues identified exhibited low similarity (Table 2). Such genetic differences between *Acinetobacter* species in key virulence attributes may help explain why some species have greater clinical impact.

Apart from genes involved in metabolism and transcription, we identified the *csu* operon as an operon found in pathogenic species of *Acinetobacter* (six *A. baumannii* strains, *A. pittii* and *A. nosocomialis*) but not in non–pathogenic species. Loss of function of this operon leads to a lack of pili–like structures on the surface of *A. baumannii* and to loss of cell attachment and biofilms on abiotic surfaces [17]. Interestingly, this operon was not shown to be important for attachment to and cytokine production by human bronchial epithelial cells [36]. We hypothesize that this operon may aid in *Acinetobacter* attachment and colonization of plastic medical devices such as ventilator tubing and catheters, with a subsequent increased risk of invasive infection. The definitive role of this operon in mammalian virulence requires further evaluation.

We observed a diverse repertoire of core metabolic genes in *A. baumannii*, which is likely to be important for its ability to survive *in vivo*, as well as in unique ecological niches of healthcare institutions. To assess the global metabolic capabilities of the *Acinetobacter* species, we used phenotype microarrays, which enabled us to assess nearly 2000 metabolic and toxic compound conditions. Overall, *A. baumannii* ATCC 19606^T^ was able to utilize nitrogen sources more effectively and was more tolerant to pH stress than *A. nosocomialis*, *A. pittii* and *A. calcoaceticus*. The differences were more marked when *A. baumannii* ATCC 19606^T^ was compared to *A. calcoaceticus*. Interestingly, *A. baumannii* and *A. pittii* were unable to utilize most of the phosphorus sources despite both strains having the necessary genetic composition for phosphate metabolism. Several studies have highlighted the key role of the Pho regulon not only in phosphate management, but also in virulence and stress responses in many bacteria [37]. Whether the inability of *A. baumannii* and *A. pittii* to utilize phosphorus is linked to expression of the Pho regulon remains a question that needs further evaluation.

Taken together, these data are hypothesis generating and provide important insights into understanding the potential differences between species of the *Acinetobacter* genus. We describe genetic and metabolic characteristics that support why some species may be more clinically important than others, and also highlight the functional significance of these differences in various virulence models. Limitations of our study are that we only analysed one strain for each of the non-*baumannii* species, and our results need confirmation using a larger set of strains. However, to our knowledge this is the first genetic and metabolic description of such a diverse range of *Acinetobacter* species. Furthermore, confirmation of our findings using targeted gene deletion and complementation is required to define the significance and role of the species-specific operons found in pathogenic versus non-pathogenic species. Finally, our study has examined the presence or absence of genes between strains; however polymorphic differences in shared genes may also contribute to phenotypic differences. A full analysis of genotype-phenotype associations will require data from many additional strains of each species. Overall, these data provide important insights into the potential differences in clinical relevance among *Acinetobacter* species.

## Materials and Methods

### Ethics Statement

The animal studies performed in this study were approved by the Leiden Experimental Animal Committee (permit number 10038) and were performed in compliance with The Experiments on Animals Act of 1996, which is the Dutch law related to the conduct of animal experiments. All efforts were undertaken to minimize suffering.

### Bacterial Strains and Culture Conditions

The 14 strains included in this study are shown in Table 1. The genomes of eight strains were sequenced in the present investigation, while for six strains, the publicly available genomes were used (Table 1). Cultures were performed at 30°C or 37°C on sheep blood agar plates (bioMérieux, Boxtel, The Netherlands) or in Luria–Bertani (LB) broth.

### Whole Genome Sequencing

Genomic DNA was extracted using the Invitrogen Easy–DNA kit (Invitrogen, CA, USA) or as described by Boom et al. [38]. Genomes were sequenced using 454 FLX pyrosequencing (Roche) with DNA standard fragment and 3 kb jumping libraries according to the manufacturer's recommendations [39]. Genomes were assembled using Newbler and annotation was performed using a combination of *ab initio* and evidence–based approaches (see Text S1).

### Phylogenetic Analysis and Comparative Genomics

Predicted proteins from each *Acinetobacter* genome were compared using an all–against–all BLAST search and *Pseudomonas aeruginosa* PAO1 was used as the outgroup. Reciprocal best blast matches (RBM), regardless of percent identity, were stored in a custom MySQL relational database to facilitate identification of orthologous groups shared by selected phylogenetic and phenotypic groups of organisms. RBM matching proteins were clustered using the Markov clustering algorithm implemented in MCL [40], and clusters with one protein per genome were defined. These represent orthologous core genes that are present as a single copy in each genome. The protein sequences for each cluster were aligned using CLUSTALW [41] and the resulting multiple sequence alignments were concatenated for tree building. A neighbour joining (NJ) tree was made using MEGA4 [42] and evaluated using 100 bootstrap replicates. The criteria used to define a putative operon were (*i*) genes were consecutive, (*ii*) genes were transcribed in the same orientation, (*iii*) the intergenic distance between the genes was no longer than 150 bp, and (*iv*) gene length was at least 450 bp [43], [44].

### Metabolic Profiling

To assess for metabolic differences between *Acinetobacter* species, we used Phenotype Microarrays (PM) as described previously by Biolog Inc. (Hayward, CA, USA) [45]. This technology uses tetrazolium violet irreversible reduction to formazan as a reporter of active metabolism. Twenty 96–well microarray plates were used (PM 1–20) comprising 1920 different metabolic and toxic compound conditions. The analysis of PM data was carried out on the raw data–set provided by Biolog Inc., obtained by three replicates of each substrate. Binary coefficients (1/0) for positive metabolism (1) or no metabolic activity (0) were attributed to each PM well and a matrix of binary vectors, each representing a single *Acinetobacte*r species, was prepared as previously described [46]. Binary data were then used to compute a similarity matrix by using Jaccard coefficient with the software PAST [47]. See text S1 for more details.

### Growth on Human Skin Equivalents

Human keratinocytes were isolated from fresh mamma reduction surplus skin and human epidermal skin constructs were generated as previously described [32], [48]. In brief, human epidermal skin constructs were incubated with 300 µl of a mid–logarithmic bacterial suspension (3×10^5^ colony forming units [CFU]/ml) at 37°C (7.3% CO_2_). After 1 h, skin constructs were washed with phosphate buffered saline (PBS) to remove non–adherent bacteria and were incubated air-exposed for an additional 23 hr and 47 hr. Two circular biopsies (4 mm in diameter) were taken from the skin, homogenized in PBS and serially diluted to determine the number of CFU. A third biopsy of each skin construct was fixed in 4% formaldehyde, dehydrated and embedded in paraffin for subsequent staining with Alcian–blue PAS (Merck, Darmstadt, Germany) for morphological analysis. Three independent experiments were performed.

### Bronchial Epithelial Cell Adhesion and Cytokine Production

Adherence of bacteria to human bronchial epithelial cells (H_292_ cells, ATCC CRL–1848, Manassas, VA, USA) and cytokine production by these cells was determined as described previously [36], [49]. In brief, H_292_ cells were incubated for 1 hr at 37°C with 1×10^8^ CFU of an overnight bacterial culture on blood agar. Bacterial adherence to H_292_ cells was quantified by light microscopy and the average number of bacteria per 100 epithelial cells was recorded. Two independent experiments were performed in duplicate. For cytokine production, H_292_ cells were washed five times after 1 hr of bacterial infection (as described above) with prewarmed PBS, and fresh RPMI medium was added. After 23 hr incubation at 37°C, supernatants were collected and stored at −20°C until determination of cytokine levels. RPMI medium alone was used as a control. Interleukin (IL)–6 and IL–8 were determined by enzyme–linked immunosorbent assays (ELISA, Biosource, CA, USA) according to the manufacturer's instructions. The lower limit of detection was 15 pg/ml for IL–6 and 7 pg/ml for IL–8. Three independent experiments were performed.

### Murine Thigh Infection Model

The survival of *Acinetobacter* strains in a mouse thigh muscle infection model was assessed as previously described [33], with modifications. Female Swiss mice (Charles River Nederland, Maastricht, The Netherlands) were made transiently neutropenic by intraperitoneal injection with cyclophosphamide (150 mg/kg body weight in 150 µl) on day 4 and 3 prior to infection. Approximately 1×10^4^ CFU (in 50 µl of saline) of a mid–logarithmic culture was injected in the right thigh muscle (three animals per strain). At 48 hr after infection, mice were sacrificed and infected thigh muscles were removed and homogenized in 1 ml PBS and viable counts were performed.

### Statistical Analysis

All data were analysed for statistical significance using the Wilcoxon rank sum test. *P* values of ≤0.05 were considered statistically significant.

## Supporting Information

Figure S1 Phylogenetic analysis of the eight sequenced strains of *Acinetobacter* species from this study.
(TIF)

Table S1 Unique genes found in pathogenic species of *Acinetobacter* (*A. baumannii* [six strains], *A. pittii* and *A. nosocomialis*) and not in other less or non-pathogenic species. Highlighted areas represent putative operons.
(DOC)

Table S2 Unique genes found in *A. baumannii* ATCC 19606^T^ compared to *A. calcoaceticus*, and their functional characterisation. Highlighted areas represent putative operons.
(DOC)

Table S3 Metabolic gains found in *A. baumannii* ATCC 19606^T^ but not in *A. calcoaceticus* using phenotypic microarrays (PM).
(DOC)

Text S1 Materials and Methods.
(DOC)
