# Supplementary figures and images for: The Success of Acinetobacter Species; Genetic, Metabolic and Virulence Attributes
Source: PLoS One. 2012 Oct 29;7(10):e46984. doi: 10.1371/journal.pone.0046984 (PMC3483291; doi:10.1371/journal.pone.0046984)

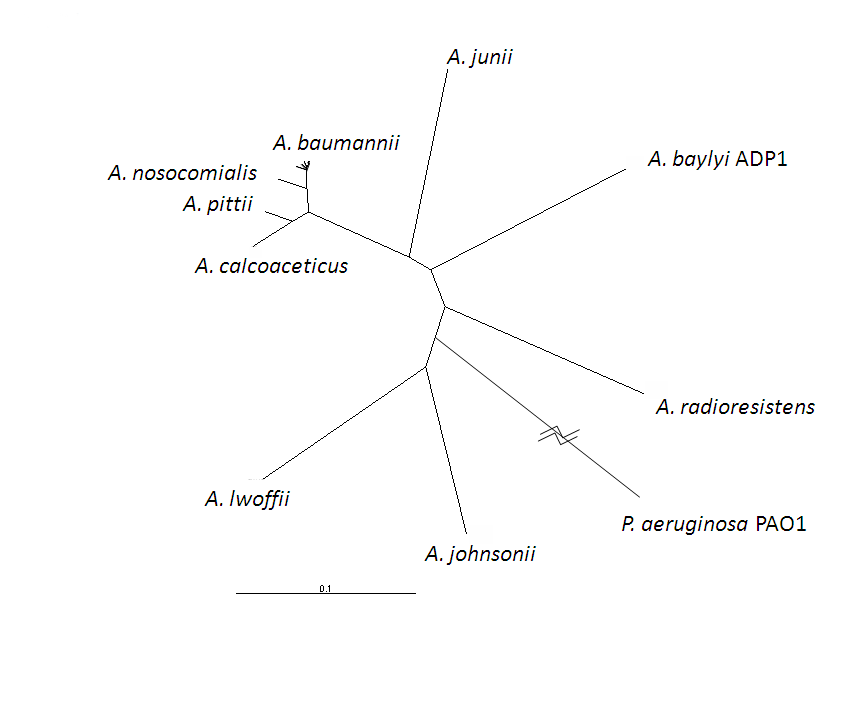

Supplement: Figure S1 — Phylogenetic analysis of the eight sequenced strains of Acinetobacter species from this study. (TIF) [file pone.0046984.s001.tif]
