# Supplementary material for: The Success of Acinetobacter Species; Genetic, Metabolic and Virulence Attributes
Source: PLoS One. 2012 Oct 29;7(10):e46984. doi: 10.1371/journal.pone.0046984 (PMC3483291; doi:10.1371/journal.pone.0046984)
Supplement: Table S1 — Unique genes found in pathogenic species of Acinetobacter (A. baumannii [six strains], A. pittii and A. nosocomialis) and not in other less or non-pathogenic species. Highlighted areas represent putative operons. (DOC) [file pone.0046984.s002.doc]

| **Supplementary Table S1.** Unique genes found in pathogenic species of *Acinetobacter* (*A. baumannii* [six strains], *A. pittii* and *A. nosocomialis*) and not in other less or non-pathogenic species. Highlighted areas represent putative operons and the two grey tones were used to help visually distinguish between consecutive operons. | | | | | |
| --- | --- | --- | --- | --- | --- |
| **ORF name from ATCC 19606** | **Gene Product** | **COG Description** | **Putative Operon** | **Homologues (nt ID %)** | |
|  |  |  |  | ***A. pittii*** | ***A. nosocomialis*** |
|  |  |  |  |  |  |
| ACIB1v1_040004 | transcriptional regulator, TetR family protein | Transcription | No | 89.62 | 90.09 |
| ACIB1v1_050001 | CsuA/B | Cell motility |  | 99.44 | 99.44 |
| ACIB1v1_050002 | CsuA | Cell motility |  | 84.07 | 85.16 |
| ACIB1v1_050003 | CsuB | Cell motility | Yes | 89.53 | 87.42 |
| ACIB1v1_050004 | CsuC | Cell motility |  | 96.34 | 94.51 |
| ACIB1v1_050005 | CsuD | Cell motility |  | 95.79 | 95.31 |
| ACIB1v1_050006 | CsuE | Cell motility |  | 95.71 | 99.28 |
| ACIB1v1_050012 | transcriptional regulator, TetR family | Transcription | No | 72.22 | 72.16 |
| ACIB1v1_050037 | acetyltransferase, gnat family | General function prediction only | No | 96 | 99.33 |
| ACIB1v1_090011 | transcriptional regulator, AsnC family | Transcription | No | 95.3 | 97.32 |
| ACIB1v1_090012 | kynureninase | Amino acid transport and metabolism |  | 90.62 | 95.91 |
| ACIB1v1_090013 | proline-specific permease ProY | Amino acid transport and metabolism | Yes | 90.17 | 96.79 |
| ACIB1v1_090014 | conserved hypothetical protein | Lipid transport and metabolism |  | 71.95 | 83.8 |
| ACIB1v1_090017 | conserved hypothetical protein | Function unknown | No | 79.61 | 89 |
| ACIB1v1_150007 | transcriptional regulator, GntR family | Transcription | Yes | 99.16 | 100 |
| ACIB1v1_150008 | dihydroxy-acid dehydratase | Carbohydrate transport and metabolism |  | 99.48 | 99.48 |
| ACIB1v1_150009 | MFS family permease | General function prediction only | Yes | 99.75 | 98.99 |
| ACIB1v1_150010 | hypothetical protein | Function unknown |  | 97.29 | 99.1 |
| ACIB1v1_150012 | conserved hypothetical protein | No COG annotation | No | 92.91 | 97.65 |
| ACIB1v1_150014 | oxidoreductase FMN-binding | Energy production and conversion | Yes | 97.82 | 97.57 |
| ACIB1v1_150015 | saccharopine dehydrogenase | Function unknown |  | 95.76 | 96.62 |
| ACIB1v1_150017 | transcriptional regulator, MerR family | Transcription | No | 91.3 | 97.83 |
| ACIB1v1_150046 | transcriptional regulator, LysR family | Transcription | No | 97.1 | 97.74 |
| ACIB1v1_150047 | hypothetical protein | Function unknown | Yes | 97.92 | 95.14 |
| ACIB1v1_150048 | hypothetical protein | Function unknown |  | 97.35 | 96.03 |
| ACIB1v1_150094 | putative acid phosphatase | General function prediction only | Yes | 97.83 | 97.2 |
| ACIB1v1_150095 | conserved hypothetical protein | No COG annotation |  | 92.99 | 94.39 |
| ACIB1v1_230050 | transcriptional regulator, LysR family | Transcription | No | 96.66 | 95.75 |
| ACIB1v1_230051 | 3-oxoacyl-[acyl-carrier-protein] reductase | Lipid transport and metabolism | No | 95.1 | 95.1 |
| ACIB1v1_240001 | conserved hypothetical protein | General function prediction only | No | 87.5 | 93.63 |
| ACIB1v1_240049 | FAD dependent oxidoreductase | Amino acid transport and metabolism | Yes | 94.79 | 92.45 |
| ACIB1v1_240050 | ABC transporter, permease | Inorganic ion transport and metabolism |  | 97.72 | 98.77 |
| ACIB1v1_240051 | ABC transporter, ATP-binding protein | Amino acid transport and metabolism |  | 95.48 | 97.59 |
| ACIB1v1_240052 | 2-aminoethylphosphonate ABC transport system, 1-aminoethylphosphonate-binding protein component | Inorganic ion transport and metabolism | Yes | 98.26 | 98.84 |
| ACIB1v1_240053 | transcriptional regulator, GntR family | Transcription |  | 97.92 | 99.58 |
| ACIB1v1_240056 | acyl carrier protein phosphodiesterase | Lipid transport and metabolism | No | 95.96 | 97.47 |
| ACIB1v1_240122 | cyanate transport protein CynX | Inorganic ion transport and metabolism | Yes | 78.77 | 90.03 |
| ACIB1v1_240123 | guanine deaminase | Nucleotide transport and metabolism |  | 80.62 | 91.25 |
| ACIB1v1_240159 | glutathione-dependent formaldehyde dehydrogenase | Amino acid transport and metabolism | No | 97.67 | 96.9 |
| ACIB1v1_240186 | 4-hydroxybenzoate transporter | Amino acid transport and metabolism | No | 94.47 | 94.47 |
| ACIB1v1_250041 | cis,cis-muconate transport protein | Amino acid transport and metabolism | No | 98.86 | 98.64 |
| ACIB1v1_250167 | conserved hypothetical protein | No COG annotation | No | 96.19 | 94.16 |
| ACIB1v1_250168 | transcriptional regulator, LysR family | Transcription | No | 98.21 | 98.57 |
| ACIB1v1_250169 | tartrate dehydrogenase | Energy production and conversion | Yes | 98.92 | 98.65 |
| ACIB1v1_250170 | betaine/choline/glycine transport protein | Cell wall/membrane/envelope biogenesis |  | 99.27 | 99.45 |
| ACIB1v1_250174 | putative dioxygenase subunit beta | Energy production and conversion |  | 95.63 | 97.27 |
| ACIB1v1_430020 | transcriptional regulator, TetR family | Transcription | No | 93.09 | 93.62 |
| ACIB1v1_460007 | Peptidase M20D, amidohydrolase | General function prediction only | No | 93.24 | 92.12 |
| ACIB1v1_490004 | TonB-dependent receptor | Inorganic ion transport and metabolism | Yes | 96.15 | 96.81 |
| ACIB1v1_490005 | aminopeptidase N | Amino acid transport and metabolism |  | 97.55 | 97.44 |
| ACIB1v1_820017 | haloacid dehydrogenase | General function prediction only | No | 93.87 | 90.57 |
|  |  |  |  |  |  |

COG, category of gene; ORF, open reading frame; nt, nucleotide
