# Supplementary material for: The Success of Acinetobacter Species; Genetic, Metabolic and Virulence Attributes
Source: PLoS One. 2012 Oct 29;7(10):e46984. doi: 10.1371/journal.pone.0046984 (PMC3483291; doi:10.1371/journal.pone.0046984)
Supplement: Table S2 — Unique genes found in A. baumannii ATCC 19606T compared to A. calcoaceticus, and their functional characterisation. Highlighted areas represent putative operons. (DOC) [file pone.0046984.s003.doc]

|  |  |  |  |  |  |
| --- | --- | --- | --- | --- | --- |
| **Supplementary Table S2.** Unique genes found in *A. baumannii* ATCC 19606^T^ compared to *A. calcoaceticus*, and their functional characterisation. Highlighted areas represent putative operons and the two grey tones were used to help visually distinguish between consecutive operons. | | | | | |
| **Gene ID** | **NCBI Accession number** | **Putative Operon ID** | **Gene Product** | **COG category** | **Homologues** |
| **ND - Not determined** | | | | | |
|  |  |  |  |  |  |
| 1 | ZP_05826619.1 |  | conserved hypothetical protein |  |  |
| 2 | ZP_05826620.1 |  | conserved hypothetical protein |  |  |
| 3 | ZP_05826621.1 |  | conserved hypothetical protein |  |  |
| 4 | ZP_05826622.1 |  | conserved hypothetical protein |  |  |
| 5 | ZP_05826623.1 |  | phage anti-repressor protein AntB | Transcription | ND |
| 6 | ZP_05826625.1 | 1 | tail tape measure protein | Function unknown | ND |
| 7 | ZP_05826626.1 |  | predicted protein |  |  |
| 8 | ZP_05826627.1 |  | predicted protein |  |  |
| 9 | ZP_05826628.1 |  | conserved hypothetical protein |  |  |
| 10 | ZP_05826629.1 |  | conserved hypothetical protein |  |  |
| 11 | ZP_05826630.1 |  | conserved hypothetical protein |  |  |
| 12 | ZP_05826631.1 |  | predicted protein |  |  |
| 13 | ZP_05826632.1 |  | conserved hypothetical protein |  |  |
| 14 | ZP_05826634.1 |  | conserved hypothetical protein |  |  |
| 15 | ZP_05826638.1 |  | conserved hypothetical protein |  |  |
| 16 | ZP_05826722.1 |  | ATPase | General function prediction only | ND |
| 17 | ZP_05826723.1 |  | transcriptional regulator GntR family | Transcription | FadR |
| 18 | ZP_05826724.1 |  | conserved hypothetical protein |  |  |
| 19 | ZP_05826725.1 |  | transcriptional regulator | Transcription | AcrR |
| 20 | ZP_05826726.1 | 2 | CsuA/B | Function unknown | ND |
| 21 | ZP_05826727.1 |  | CsuA |  | ND |
| 22 | ZP_05826728.1 |  | CsuB | Function unknown | ND |
| 23 | ZP_05826729.1 |  | CsuC | Cell motility and secretion | FimC |
| 24 | ZP_05826730.1 |  | CsuD | Cell motility and secretion | FimD |
| 25 | ZP_05826731.1 |  | CsuE | Function unknown | ND |
| 26 | ZP_05826732.1 |  | conserved hypothetical protein |  |  |
| 27 | ZP_05826736.1 |  | acetyltransferase | Translation, ribosomal structure and biogenesis | RimL |
| 28 | ZP_05826737.1 |  | TetR family transcriptional regulator | Transcription | AcrR |
| 29 | ZP_05826738.1 |  | DMT family permease | Carbohydrate transport and metabolism | RhaT |
| 30 | ZP_05826762.1 |  | histone acetyltransferase HPA2 | Amino acid transport and metabolism | ArgA |
| 31 | ZP_05826811.1 |  | acyl-CoA ligase | Lipid metabolism | Acs |
| 32 | ZP_05826875.1 |  | conserved hypothetical protein |  |  |
| 33 | ZP_05826877.1 |  | conserved hypothetical protein |  |  |
| 34 | ZP_05826878.1 |  | TetR family transcriptional regulator | Transcription | AcrR |
| 35 | ZP_05826882.1 |  | conserved hypothetical protein |  |  |
| 36 | ZP_05826884.1 |  | conserved hypothetical protein |  |  |
| 37 | ZP_05826887.1 |  | OmpW family protein | Cell envelope biogenesis, outer membrane | OmpW |
| 38 | ZP_05826895.1 |  | conserved hypothetical protein |  |  |
| 39 | ZP_05826896.1 | 3 | magnesium-translocating P-type ATPase | Inorganic ion transport and metabolism | MgtA |
| 40 | ZP_05826897.1 |  | mgtC | Function unknown | SapB |
| 41 | ZP_05826921.1 |  | transcriptional regulator | Transcription | AcrR |
| 42 | ZP_05826922.1 |  | conserved hypothetical protein |  |  |
| 43 | ZP_05826937.1 |  | anaerobic dehydrogenase | Energy production and conversion | BisC |
| 44 | ZP_05826952.1 |  | conserved hypothetical protein |  |  |
| 45 | ZP_05826984.1 |  | transcriptional regulator | Transcription | Lrp |
| 46 | ZP_05826985.1 | 4 | kynureninase | Amino acid transport and metabolism | ND |
| 47 | ZP_05826986.1 |  | GABA permease | Amino acid transport and metabolism | AnsP |
| 48 | ZP_05826987.1 |  | esterase/lipase | Lipid metabolism | Aes |
| 49 | ZP_05826996.1 |  | conserved hypothetical protein |  |  |
| 50 | ZP_05827026.1 |  | ferrichrome-iron receptor protein | Inorganic ion transport and metabolism | Fiu |
| 51 | ZP_05827035.1 |  | conserved hypothetical protein |  |  |
| 52 | ZP_05827057.1 |  | acyl CoA:acetate/3-ketoacid CoA transferase | Lipid metabolism | AtoA |
| 53 | ZP_05827058.1 |  | beta-ketoadipyl CoA thiolase | Lipid metabolism | PaaJ |
| 54 | ZP_05827081.1 |  | conserved hypothetical protein |  |  |
| 55 | ZP_05827096.1 |  | peptidase S45 | General function prediction only | ND |
| 56 | ZP_05827099.1 |  | acyl CoA:acetate/3-ketoacid CoA transferase | Lipid metabolism | AtoA |
| 57 | ZP_05827100.1 |  | 3-oxoadipate CoA-transferase subunit A | Lipid metabolism | AtoD |
| 58 | ZP_05827106.1 |  | transcriptional regulator GntR family | Transcription | FadR |
| 59 | ZP_05827107.1 |  | dihydroxy-acid dehydratase | Amino acid transport and metabolism | IlvD |
| 60 | ZP_05827108.1 | 5 | 4-hydroxyphenylacetate permease | Carbohydrate transport and metabolism | UhpC |
| 61 | ZP_05827109.1 |  | sugar transporter | Function unknown | GguC |
| 62 | ZP_05827110.1 |  | 2-hydroxymuconic semialdehyde dehydrogenase | Energy production and conversion | PutA |
| 63 | ZP_05827111.1 |  | conserved hypothetical protein |  |  |
| 64 | ZP_05827113.1 | 6 | oxidoreductase FMN-binding | Energy production and conversion | NemA |
| 65 | ZP_05827114.1 |  | saccharopine dehydrogenase | Function unknown | ND |
| 66 | ZP_05827115.1 |  | ThiJ/PfpI domain-containing protein | General function prediction only | ThiJ |
| 67 | ZP_05827116.1 |  | Cu(I)-responsive transcriptional regulator | Transcription | SoxR |
| 68 | ZP_05827131.1 |  | conserved hypothetical protein |  |  |
| 69 | ZP_05827132.1 |  | bacterial regulatory protein | Transcription | AcrR |
| 70 | ZP_05827135.1 |  | predicted protein | Function unknown | ND |
| 71 | ZP_05827136.1 |  | predicted protein |  |  |
| 72 | ZP_05827137.1 |  | conserved hypothetical protein |  |  |
| 73 | ZP_05827138.1 | 7 | 4-hydroxyphenylacetate permease | Carbohydrate transport and metabolism | UhpC |
| 74 | ZP_05827139.1 |  | amidohydrolase | General function prediction only | ND |
| 75 | ZP_05827140.1 |  | dicarboxylate carrier protein MatC family protein | Inorganic ion transport and metabolism | CitT |
| 76 | ZP_05827141.1 |  | transcriptional regulator | Transcription | PhnF |
| 77 | ZP_05827142.1 |  | senescence marker protein-30 | Carbohydrate transport and metabolism | ND |
| 78 | ZP_05827143.1 |  | major facilitator superfamily transporter permease | Carbohydrate transport and metabolism | UhpC |
| 79 | ZP_05827144.1 |  | transcriptional regulator | Transcription | LysR |
| 80 | ZP_05827145.1 | 8 | conserved hypothetical protein | Function unknown | ND |
| 81 | ZP_05827146.1 |  | conserved hypothetical protein | Function unknown | ND |
| 82 | ZP_05827150.1 | 9 | 2-hydroxymuconic semialdehyde dehydrogenase | Energy production and conversion | PutA |
| 83 | ZP_05827151.1 |  | dihydroxy-acid dehydratase | Amino acid transport and metabolism | IlvD |
| 84 | ZP_05827152.1 |  | fumarylacetoacetate hydrolase | General function prediction only | ND |
| 85 | ZP_05827153.1 |  | transcriptional regulator LysR family | Transcription | LysR |
| 86 | ZP_05827154.1 | 10 | nucleoside-diphosphate-sugar epimerase | Cell envelope biogenesis, outer membrane | WcaG |
| 87 | ZP_05827155.1 |  | 4-hydroxyphenylacetate permease | Carbohydrate transport and metabolism | UhpC |
| 88 | ZP_05827156.1 |  | 2-hydroxy-3-oxopropionate reductase | Lipid metabolism | MmsB |
| 89 | ZP_05827158.1 | 11 | cupin 2 | Function unknown | ND |
| 90 | ZP_05827159.1 |  | 4-carboxymuconolactone decarboxylase domain-containing protein | Function unknown | ND |
| 91 | ZP_05827161.1 |  | conserved hypothetical protein |  |  |
| 92 | ZP_05827172.1 |  | predicted protein |  |  |
| 93 | ZP_05827174.1 |  | predicted protein |  |  |
| 94 | ZP_05827175.1 |  | predicted protein |  |  |
| 95 | ZP_05827176.1 |  | predicted protein |  |  |
| 96 | ZP_05827177.1 |  | restriction endonuclease |  |  |
| 97 | ZP_05827178.1 |  | type II restriction-modification system restriction subunit | Defense mechanisms | McrB |
| 98 | ZP_05827180.1 |  | T/G mismatch-specific endonuclease | DNA replication, recombination, and repair | Vsr |
| 99 | ZP_05827181.1 | 12 | conserved hypothetical protein |  |  |
| 100 | ZP_05827182.1 |  | conserved hypothetical protein | Function unknown | ND |
| 101 | ZP_05827183.1 |  | predicted protein |  |  |
| 102 | ZP_05827191.1 | 13 | acid phosphatase | General function prediction only | SurE |
| 103 | ZP_05827192.1 |  | signal peptide containing protein |  |  |
| 104 | ZP_05827195.1 |  | TetR family transcriptional regulator | Transcription | AcrR |
| 105 | ZP_05827196.1 |  | predicted protein |  |  |
| 106 | ZP_05827197.1 |  | type III restriction enzyme | Transcription | SSL2 |
| 107 | ZP_05827199.1 |  | conserved hypothetical protein |  |  |
| 108 | ZP_05827200.1 |  | RNA 2'-phosphotransferase | Translation, ribosomal structure and biogenesis | KptA |
| 109 | ZP_05827202.1 | 14 | AttS | Amino acid transport and metabolism | DAP2 |
| 110 | ZP_05827203.1 |  | cis-2,3-dihydrobiphenyl-2,3-diol dehydrogenase | Secondary metabolites biosynthesis, transport, and catabolism | FabG |
| 111 | ZP_05827204.1 |  | 2-hydroxycyclohexanecarboxyl-CoA dehydrogenase | General function prediction only | ND |
| 112 | ZP_05827205.1 |  | X-Pro dipeptidyl-peptidase family protein | Amino acid transport and metabolism | DAP2 |
| 113 | ZP_05827206.1 |  | AraC family transcriptional regulator | Transcription | AraC |
| 114 | ZP_05827255.1 |  | conserved hypothetical protein |  |  |
| 115 | ZP_05827256.1 |  | transcriptional regulator | Transcription | AcrR |
| 116 | ZP_05827257.1 |  | conserved hypothetical protein | Function unknown | ND |
| 117 | ZP_05827258.1 |  | conserved hypothetical protein |  |  |
| 118 | ZP_05827259.1 |  | predicted protein |  |  |
| 119 | ZP_05827271.1 |  | conserved hypothetical protein | Function unknown | ND |
| 120 | ZP_05827272.1 | 15 | HEAT repeat-containing protein | Energy production and conversion | ND |
| 121 | ZP_05827273.1 |  | 2-aminoethylphosphonate ABC transport system, ATP-binding component PhnT | Inorganic ion transport and metabolism | TauB |
| 122 | ZP_05827274.1 |  | nitrate ABC transporter, permease | Inorganic ion transport and metabolism | TauC |
| 123 | ZP_05827275.1 |  | ABC-type nitrate/sulfonate/bicarbonate transport system | Inorganic ion transport and metabolism | TauA |
| 124 | ZP_05827276.1 |  | adenylylsulfate reductase beta subunit | General function prediction only | ND |
| 125 | ZP_05827277.1 |  | succinate dehydrogenase/fumarate reductase | Energy production and conversion | SdhA |
| 126 | ZP_05827278.1 |  | transcriptional regulator | Transcription | PhnF |
| 127 | ZP_05827281.1 |  | transcriptional regulator | Transcription | ND |
| 128 | ZP_05827319.1 | 16 | ABC transporter periplasmic substrate-binding protein | Defense mechanisms | ND |
| 129 | ZP_05827320.1 |  | ABC-type multidrug transport system | Defense mechanisms | ND |
| 130 | ZP_05827321.1 |  | ABC transporter membrane protein | Energy production and conversion | NatB |
| 131 | ZP_05827322.1 |  | PltJ | Defense mechanisms | CcmA |
| 132 | ZP_05827323.1 |  | secretion protein HlyD | Defense mechanisms | EmrA |
| 133 | ZP_05827324.1 |  | TetR family transcriptional regulator | Transcription | AcrR |
| 134 | ZP_05827338.1 |  | conserved hypothetical protein |  |  |
| 135 | ZP_05827340.1 |  | siderophore biosynthesis protein | Translation, ribosomal structure and biogenesis | RimL |
| 136 | ZP_05827341.1 |  | conserved hypothetical protein |  |  |
| 137 | ZP_05827342.1 |  | PepSY-associated TM helix family protein | Function unknown | PiuB |
| 138 | ZP_05827343.1 |  | conserved hypothetical protein |  |  |
| 139 | ZP_05827344.1 | 17 | ferric aerobactin receptor | Inorganic ion transport and metabolism | CirA |
| 140 | ZP_05827345.1 |  | dimethylmenaquinone methyltransferase | Coenzyme metabolism | MenG |
| 141 | ZP_05827346.1 |  | conserved hypothetical protein |  |  |
| 142 | ZP_05827349.1 |  | MFS superfamily multidrug resistance protein | Carbohydrate transport and metabolism | AraJ |
| 143 | ZP_05827350.1 |  | L-lysine 6-monooxygenase | Secondary metabolites biosynthesis, transport, and catabolism | rhbE (iucD) |
| 144 | ZP_05827351.1 |  | siderophore synthetase component | Secondary metabolites biosynthesis, transport, and catabolism | RhbC (iucA) |
| 145 | ZP_05827394.1 |  | acetyltransferase |  |  |
| 146 | ZP_05827464.1 |  | conserved hypothetical protein |  |  |
| 147 | ZP_05827482.1 |  | conserved hypothetical protein |  |  |
| 148 | ZP_05827483.1 |  | integrase |  |  |
| 149 | ZP_05827484.1 |  | conserved hypothetical protein |  |  |
| 150 | ZP_05827485.1 |  | ATPase | Replication, recombination, and repair | ND |
| 151 | ZP_05827486.1 | 18 | conserved hypothetical protein |  |  |
| 152 | ZP_05827487.1 |  | yeeC |  |  |
| 153 | ZP_05827488.1 |  | conserved hypothetical protein |  |  |
| 154 | ZP_05827489.1 |  | yeeB | Transcription | SSL2 |
| 155 | ZP_05827490.1 | 19 | yeeA | Defense mechanisms | ND |
| 156 | ZP_05827491.1 |  | predicted protein |  |  |
| 157 | ZP_05827492.1 |  | predicted protein |  |  |
| 158 | ZP_05827493.1 | 20 | transposase subunit A | DNA replication, recombination, and repair | ND |
| 159 | ZP_05827494.1 |  | Y4tA | DNA replication, recombination, and repair | DnaC |
| 160 | ZP_05827495.1 |  | predicted protein |  |  |
| 161 | ZP_05827558.1 |  | conserved hypothetical protein |  |  |
| 162 | ZP_05827573.1 |  | D-serine deaminase transcriptional activator | Transcription | LysR |
| 163 | ZP_05827574.1 |  | anti-sigm factor ChrR | Transcription | ChrR |
| 164 | ZP_05827584.1 |  | LysR family transcriptional regulator | Transcription | LysR |
| 165 | ZP_05827585.1 |  | 3-oxoacyl-[acyl-carrier-protein] reductase | Secondary metabolites biosynthesis, transport, and catabolism | FabG |
| 166 | ZP_05827605.1 |  | predicted protein |  |  |
| 167 | ZP_05827606.1 |  | conserved hypothetical protein |  |  |
| 168 | ZP_05827607.1 |  | conserved hypothetical protein |  |  |
| 169 | ZP_05827608.1 |  | 2-hydroxycyclohexanecarboxyl-CoA dehydrogenase | Secondary metabolites biosynthesis, transport, and catabolism | FabG |
| 170 | ZP_05827612.1 |  | predicted protein |  |  |
| 171 | ZP_05827614.1 |  | conserved hypothetical protein |  |  |
| 172 | ZP_05827652.1 | 21 | phosphonoacetaldehyde hydrolase | General function prediction only | ND |
| 173 | ZP_05827653.1 |  | 2-aminoethylphosphonate-pyruvate transaminase | Amino acid transport and metabolism | ND |
| 174 | ZP_05827654.1 |  | monomeric sarcosine oxidase(MSOX) | Amino acid transport and metabolism | DadA |
| 175 | ZP_05827655.1 |  | ABC-type Fe3+ transport system permease component | Inorganic ion transport and metabolism | ThiP |
| 176 | ZP_05827656.1 |  | 2-aminoethylphosphonate ABC transport system, ATP-binding component PhnT | Amino acid transport and metabolism | PotA |
| 177 | ZP_05827657.1 |  | ABC transporter periplasmic substrate-binding protein | Inorganic ion transport and metabolism | AfuA |
| 178 | ZP_05827658.1 |  | transcriptional regulator | Transcription | PhnF |
| 179 | ZP_05827661.1 |  | phosphodiesterase | Lipid metabolism | AcpD |
| 180 | ZP_05827667.1 |  | conserved hypothetical protein |  |  |
| 181 | ZP_05827675.1 | 22 | cytochrome b561 family protein | Energy production and conversion | CybB |
| 182 | ZP_05827676.1 |  | catalase | Inorganic ion transport and metabolism | KatE |
| 183 | ZP_05827677.1 | 23 | 4-hydroxyphenylacetate permease | Carbohydrate transport and metabolism | UhpC |
| 184 | ZP_05827678.1 |  | 3-hydroxy-3-methylglutaryl-CoA lyase | Amino acid transport and metabolism | LeuA |
| 185 | ZP_05827679.1 |  | formyl-CoA transferase | Energy production and conversion | CaiB |
| 186 | ZP_05827680.1 |  | transcriptional regulator | Transcription | LysR |
| 187 | ZP_05827683.1 |  | conserved hypothetical protein |  |  |
| 188 | ZP_05827684.1 |  | bacterial regulatory protein | Transcription | AcrR |
| 189 | ZP_05827685.1 |  | conserved hypothetical protein | DNA replication, recombination, and repair | ND |
| 190 | ZP_05827686.1 |  | predicted protein |  |  |
| 191 | ZP_05827687.1 |  | predicted protein | General function prediction only | ND |
| 192 | ZP_05827688.1 |  | conserved hypothetical protein |  |  |
| 193 | ZP_05827689.1 |  | predicted protein |  |  |
| 194 | ZP_05827705.1 |  | amino acid transporter | Amino acid transport and metabolism | PotE |
| 195 | ZP_05827706.1 |  | 2-hydroxymuconic semialdehyde dehydrogenase | Energy production and conversion | PutA |
| 196 | ZP_05827707.1 |  | major facilitator superfamily transporter permease | Carbohydrate transport and metabolism | MelB |
| 197 | ZP_05827708.1 | 24 | AraC-type DNA-binding domain-containing protein | Transcription | AraC |
| 198 | ZP_05827709.1 |  | pyridine nucleotide-disulphide oxidoreductase | General function prediction only | HcaD |
| 199 | ZP_05827710.1 |  | ferredoxin | General function prediction only | ND |
| 200 | ZP_05827711.1 | 25 | glycine oxidase ThiO | Amino acid transport and metabolism | DadA |
| 201 | ZP_05827712.1 |  | proline racemase | Amino acid transport and metabolism | ND |
| 202 | ZP_05827713.1 |  | GntR family transcriptional regulator | Transcription | GntR |
| 203 | ZP_05827714.1 |  | DMT family permease | Amino acid transport and metabolism | rhtA |
| 204 | ZP_05827715.1 |  | dihydrodipicolinate synthetase | Amino acid transport and metabolism | DapA |
| 205 | ZP_05827722.1 | 26 | cyanate permease | Inorganic ion transport and metabolism | CynX |
| 206 | ZP_05827723.1 |  | guanine deaminase | Nucleotide transport and metabolism | CumB |
| 207 | ZP_05827724.1 |  | ankyrin repeat-containing protein | General function prediction only | Arp |
| 208 | ZP_05827750.1 |  | conserved hypothetical protein | DNA replication, recombination, and repair | ND |
| 209 | ZP_05827751.1 |  | conserved hypothetical protein |  |  |
| 210 | ZP_05827757.1 |  | formaldehyde dehydrogenase, glutathione-independent | Amino acid transport and metabolism | Tdh |
| 211 | ZP_05827760.1 |  | predicted protein |  |  |
| 212 | ZP_05827761.1 | 27 | predicted protein |  |  |
| 213 | ZP_05827762.1 |  | conserved hypothetical protein |  |  |
| 214 | ZP_05827763.1 |  | FhaB protein |  | FhaB |
| 215 | ZP_05827764.1 |  | hemolysin activation/secretion protein | Intracellular trafficking and secretion | FhaC |
| 216 | ZP_05827773.1 |  | beta-lactamase class A | Defense mechanisms | PenP |
| 217 | ZP_05827775.1 |  | acetyltransferase family protein |  |  |
| 218 | ZP_05827780.1 |  | transcriptional regulator | Transcription | SoxR |
| 219 | ZP_05827782.1 |  | major facilitator superfamily transporter permease | Carbohydrate transport and metabolism | AraJ |
| 220 | ZP_05827783.1 |  | TetR family transcriptional regulator | Transcription | AcrR |
| 221 | ZP_05827784.1 |  | conserved hypothetical protein |  |  |
| 222 | ZP_05827787.1 |  | predicted protein |  |  |
| 223 | ZP_05827788.1 |  | inner membrane transporter YbiF | Amino acid transport and metabolism | rhtA |
| 224 | ZP_05827790.1 |  | conserved hypothetical protein |  |  |
| 225 | ZP_05827838.1 |  | major facilitator superfamily transporter cis,cis-muconate transporter | Carbohydrate transport and metabolism | UhpC |
| 226 | ZP_05827862.1 |  | conserved hypothetical protein |  |  |
| 227 | ZP_05827961.1 |  | conserved hypothetical protein |  |  |
| 228 | ZP_05827962.1 |  | transcriptional regulator | Transcription | LysR |
| 229 | ZP_05827963.1 | 28 | tartrate dehydrogenase | Amino acid transport and metabolism | LeuB |
| 230 | ZP_05827964.1 |  | betaine/choline/glycine transporter | Cell envelope biogenesis, outer membrane | BetT |
| 231 | ZP_05827967.1 |  | betaine aldehyde dehydrogenase | Energy production and conversion | PutA |
| 232 | ZP_05827968.1 |  | dioxygenase beta subunit | Energy production and conversion | Hmp |
| 233 | ZP_05827972.1 |  | pirin family protein | General function prediction only | ND |
| 234 | ZP_05827979.1 |  | predicted protein | Translation, ribosomal structure and biogenesis | TufB |
| 235 | ZP_05827986.1 |  | conserved hypothetical protein |  |  |
| 236 | ZP_05828022.1 |  | integrase | DNA replication, recombination, and repair | XerD |
| 237 | ZP_05828023.1 |  | predicted protein |  |  |
| 238 | ZP_05828024.1 |  | conserved hypothetical protein |  |  |
| 239 | ZP_05828025.1 |  | prophage antirepressor |  |  |
| 240 | ZP_05828026.1 |  | gp54 protein | Transcription | ND |
| 241 | ZP_05828027.1 |  | conserved hypothetical protein |  |  |
| 242 | ZP_05828028.1 |  | conserved hypothetical protein |  |  |
| 243 | ZP_05828029.1 |  | conserved hypothetical protein |  |  |
| 244 | ZP_05828030.1 |  | inner membrane protein | DNA replication, recombination, and repair | ND |
| 245 | ZP_05828031.1 |  | predicted protein | DNA replication, recombination, and repair | Ssb |
| 246 | ZP_05828032.1 |  | conserved hypothetical protein |  |  |
| 247 | ZP_05828033.1 |  | conserved hypothetical protein |  |  |
| 248 | ZP_05828034.1 |  | predicted protein |  |  |
| 249 | ZP_05828035.1 |  | conserved hypothetical protein |  |  |
| 250 | ZP_05828036.1 |  | nucleoid-associated protein | General function prediction only | ND |
| 251 | ZP_05828037.1 |  | conserved hypothetical protein |  |  |
| 252 | ZP_05828038.1 |  | predicted protein |  |  |
| 253 | ZP_05828039.1 |  | predicted protein |  |  |
| 254 | ZP_05828040.1 |  | predicted protein |  |  |
| 255 | ZP_05828041.1 |  | predicted protein |  |  |
| 256 | ZP_05828042.1 |  | conserved hypothetical protein |  |  |
| 257 | ZP_05828043.1 |  | predicted protein |  |  |
| 258 | ZP_05828044.1 | 29 | CRISPR-associated protein cas1 | Defense mechanisms | ND |
| 259 | ZP_05828045.1 |  | conserved hypothetical protein |  |  |
| 260 | ZP_05828046.1 |  | CRISPR-associated protein | RNA processing and modification | PRP8 |
| 261 | ZP_05828047.1 |  | conserved hypothetical protein |  |  |
| 262 | ZP_05828048.1 |  | conserved hypothetical protein |  |  |
| 263 | ZP_05828049.1 |  | conserved hypothetical protein |  |  |
| 264 | ZP_05828050.1 |  | predicted protein |  |  |
| 265 | ZP_05828051.1 |  | CRISPR-associated helicase cas3 | Defense mechanisms | ND |
| 266 | ZP_05828052.1 |  | predicted protein |  |  |
| 267 | ZP_05828053.1 |  | predicted protein |  |  |
| 268 | ZP_05828054.1 | 30 | conserved hypothetical protein | Function unknown | ND |
| 269 | ZP_05828055.1 |  | gp29 | Function unknown | ND |
| 270 | ZP_05828056.1 |  | conserved hypothetical protein |  |  |
| 271 | ZP_05828057.1 |  | predicted protein |  |  |
| 272 | ZP_05828058.1 |  | predicted protein |  |  |
| 273 | ZP_05828059.1 |  | predicted protein |  |  |
| 274 | ZP_05828060.1 |  | predicted protein |  |  |
| 275 | ZP_05828067.1 |  | rhs element Vgr family protein | Function unknown | VgrG |
| 276 | ZP_05828068.1 |  | predicted protein |  |  |
| 277 | ZP_05828069.1 |  | predicted protein |  |  |
| 278 | ZP_05828070.1 |  | conserved hypothetical protein | Function unknown | ND |
| 279 | ZP_05828090.1 |  | conserved hypothetical protein |  |  |
| 280 | ZP_05828118.1 |  | transcriptional regulatory protein | Transcription | LysR |
| 281 | ZP_05828119.1 | 31 | 3-oxoacyl-[acyl-carrier-protein] reductase | Secondary metabolites biosynthesis, transport, and catabolism | FabG |
| 282 | ZP_05828120.1 |  | 4-hydroxyphenylacetate permease | Carbohydrate transport and metabolism | UhpC |
| 283 | ZP_05828121.1 |  | transketolase | Carbohydrate transport and metabolism | ND |
| 284 | ZP_05828122.1 |  | transketolase | Carbohydrate transport and metabolism | ND |
| 285 | ZP_05828124.1 |  | conserved hypothetical protein |  |  |
| 286 | ZP_05828131.1 |  | conserved hypothetical protein |  |  |
| 287 | ZP_05828164.1 |  | conserved hypothetical protein |  |  |
| 288 | ZP_05828178.1 |  | predicted protein |  |  |
| 289 | ZP_05828185.1 |  | ATP-dependent protease La | Posttranslational modification, protein turnover, chaperones | Lon |
| 290 | ZP_05828187.1 | 32 | autotransporter adhesin | Function unknown | ND |
| 291 | ZP_05828188.1 |  | small protein A | Cell envelope biogenesis, outer membrane | OmpA |
| 292 | ZP_05828193.1 |  | conserved hypothetical protein |  |  |
| 293 | ZP_05828194.1 |  | conserved hypothetical protein |  |  |
| 294 | ZP_05828195.1 |  | conserved hypothetical protein |  |  |
| 295 | ZP_05828196.1 |  | conserved hypothetical protein |  |  |
| 296 | ZP_05828197.1 |  | conserved hypothetical protein |  |  |
| 297 | ZP_05828198.1 |  | conserved hypothetical protein |  |  |
| 298 | ZP_05828199.1 |  | conserved hypothetical protein |  |  |
| 299 | ZP_05828200.1 |  | conserved hypothetical protein |  |  |
| 300 | ZP_05828202.1 |  | conserved hypothetical protein |  |  |
| 301 | ZP_05828203.1 |  | conserved hypothetical protein |  |  |
| 302 | ZP_05828204.1 |  | conserved hypothetical protein |  |  |
| 303 | ZP_05828205.1 |  | conserved hypothetical protein |  |  |
| 304 | ZP_05828206.1 |  | conserved hypothetical protein |  |  |
| 305 | ZP_05828208.1 |  | conserved hypothetical protein |  |  |
| 306 | ZP_05828209.1 |  | conserved hypothetical protein |  |  |
| 307 | ZP_05828210.1 |  | conserved hypothetical protein |  |  |
| 308 | ZP_05828211.1 |  | conserved hypothetical protein |  |  |
| 309 | ZP_05828212.1 |  | conserved hypothetical protein |  |  |
| 310 | ZP_05828213.1 |  | conserved hypothetical protein |  |  |
| 311 | ZP_05828214.1 |  | conserved hypothetical protein |  |  |
| 312 | ZP_05828215.1 |  | conserved hypothetical protein |  |  |
| 313 | ZP_05828217.1 |  | conserved hypothetical protein |  |  |
| 314 | ZP_05828218.1 |  | conserved hypothetical protein |  |  |
| 315 | ZP_05828220.1 |  | conserved hypothetical protein |  |  |
| 316 | ZP_05828223.1 |  | conserved hypothetical protein |  |  |
| 317 | ZP_05828224.1 | 33 | conserved hypothetical protein |  |  |
| 318 | ZP_05828226.1 |  | conserved hypothetical protein |  |  |
| 319 | ZP_05828227.1 |  | phage protein | Function unknown | ND |
| 320 | ZP_05828228.1 |  | PBSX family phage terminase, large subunit |  |  |
| 321 | ZP_05828232.1 |  | conserved hypothetical protein |  |  |
| 322 | ZP_05828233.1 |  | conserved hypothetical protein |  |  |
| 323 | ZP_05828239.1 |  | conserved hypothetical protein |  |  |
| 324 | ZP_05828240.1 |  | conserved hypothetical protein |  |  |
| 325 | ZP_05828245.1 |  | conserved hypothetical protein | Signal transduction mechanisms | ND |
| 326 | ZP_05828246.1 |  | conserved hypothetical protein |  |  |
| 327 | ZP_05828266.1 |  | predicted protein |  |  |
| 328 | ZP_05828275.1 |  | conserved hypothetical protein |  |  |
| 329 | ZP_05828310.1 |  | conserved hypothetical protein |  |  |
| 330 | ZP_05828312.1 |  | YjgF family translation initiation inhibitor | Translation, ribosomal structure and biogenesis | TdcF |
| 331 | ZP_05828385.1 |  | conserved hypothetical protein |  |  |
| 332 | ZP_05828386.1 |  | phage replication protein | DNA replication, recombination, and repair | ND |
| 333 | ZP_05828387.1 |  | predicted protein |  |  |
| 334 | ZP_05828388.1 |  | predicted protein |  |  |
| 335 | ZP_05828389.1 |  | predicted protein |  |  |
| 336 | ZP_05828390.1 |  | conserved hypothetical protein |  |  |
| 337 | ZP_05828391.1 |  | predicted protein |  |  |
| 338 | ZP_05828392.1 |  | zonular occludens toxin |  |  |
| 339 | ZP_05828393.1 |  | pilin inverting protein | DNA replication, recombination, and repair | ND |
| 340 | ZP_05828444.1 |  | transmembrane protein |  |  |
| 341 | ZP_05828457.1 |  | conserved hypothetical protein |  |  |
| 342 | ZP_05828544.1 |  | conserved hypothetical protein |  |  |
| 343 | ZP_05828571.1 |  | predicted protein | Translation, ribosomal structure and biogenesis | TufB |
| 344 | ZP_05828631.1 |  | conserved hypothetical protein |  |  |
| 345 | ZP_05828655.1 |  | aminoglycoside 2'-N-acetyltransferase(AAC(2')-Ib) | General function prediction only | Eis |
| 346 | ZP_05828696.1 |  | conserved hypothetical protein |  |  |
| 347 | ZP_05828697.1 |  | non-ribosomal peptide synthetase module | Secondary metabolites biosynthesis, transport, and catabolism | EntF |
| 348 | ZP_05828698.1 |  | delta-aminolevulinic acid dehydratase |  |  |
| 349 | ZP_05828699.1 |  | predicted protein |  |  |
| 350 | ZP_05828700.1 | 34 | conserved hypothetical protein |  |  |
| 351 | ZP_05828701.1 |  | conserved hypothetical protein |  |  |
| 352 | ZP_05828702.1 |  | transcriptional regulator | Transcription | AcrR |
| 353 | ZP_05828705.1 |  | TetR family transcriptional regulator | Transcription | AcrR |
| 354 | ZP_05828708.1 |  | conserved hypothetical protein |  |  |
| 355 | ZP_05828715.1 |  | conserved hypothetical protein |  |  |
| 356 | ZP_05828720.1 |  | ADP-ribosylglycohydrolase superfamily | Posttranslational modification, protein turnover, chaperones | DraG |
| 357 | ZP_05828727.1 |  | 4-amino-4-deoxy-L-arabinose transferase | Cell envelope biogenesis, outer membrane | ArnT |
| 358 | ZP_05828728.1 |  | conserved hypothetical protein |  |  |
| 359 | ZP_05828729.1 | 35 | glycosyltransferase | Cell envelope biogenesis, outer membrane | WcaA |
| 360 | ZP_05828730.1 |  | conserved hypothetical protein | Function unknown | ND |
| 361 | ZP_05828735.1 |  | predicted protein |  |  |
| 362 | ZP_05828737.1 |  | conserved hypothetical protein |  |  |
| 363 | ZP_05828771.1 |  | conserved hypothetical protein |  |  |
| 364 | ZP_05828772.1 |  | conserved hypothetical protein |  |  |
| 365 | ZP_05828810.1 |  | chaperone DnaK | Posttranslational modification, protein turnover, chaperones | DnaK |
| 366 | ZP_05828811.1 |  | conserved hypothetical protein |  |  |
| 367 | ZP_05828844.1 |  | conserved hypothetical protein |  |  |
| 368 | ZP_05828845.1 |  | conserved hypothetical protein |  |  |
| 369 | ZP_05828864.1 |  | NAD(P)H oxidoreductase | General function prediction only | MdaB |
| 370 | ZP_05828865.1 |  | transcriptional regulator lysR family | Transcription | LysR |
| 371 | ZP_05828903.1 |  | conserved hypothetical protein |  |  |
| 372 | ZP_05828904.1 |  | conserved hypothetical protein |  |  |
| 373 | ZP_05828905.1 |  | conserved hypothetical protein |  |  |
| 374 | ZP_05828910.1 |  | vulnibactin utilization protein viuB | Inorganic ion transport and metabolism | ViuB |
| 375 | ZP_05828911.1 |  | non-ribosomal peptide synthetase | Secondary metabolites biosynthesis, transport, and catabolism | EntF |
| 376 | ZP_05828912.1 |  | non-ribosomal peptide synthetase | Secondary metabolites biosynthesis, transport, and catabolism | EntF |
| 377 | ZP_05828913.1 |  | conserved hypothetical protein |  |  |
| 378 | ZP_05828914.1 | 36 | BauD | Inorganic ion transport and metabolism | BauD (CeuB or fatD) |
| 379 | ZP_05828915.1 |  | BauC | Inorganic ion transport and metabolism | BauC (CeuC or fatC) |
| 380 | ZP_05828916.1 |  | phosphonate ABC transporter, ATP-binding protein | Inorganic ion transport and metabolism | BauE (CeuD or fatE) |
| 381 | ZP_05828917.1 |  | BauB | Inorganic ion transport and metabolism | BauB (CeuA or fatB) |
| 382 | ZP_05828918.1 |  | ferric anguibactin receptor | Inorganic ion transport and metabolism | BauA (Fiu or fatA) |
| 383 | ZP_05828919.1 | 37 | BasC | Secondary metabolites biosynthesis, transport, and catabolism | IucD (basC) |
| 384 | ZP_05828920.1 |  | BasD | Secondary metabolites biosynthesis, transport, and catabolism | EntF (basD) |
| 385 | ZP_05828922.1 | 38 | acinetobactin biosynthesis protein | Amino acid transport and metabolism | basE |
| 386 | ZP_05828923.1 |  | isochorismate hydrolase | Secondary metabolites biosynthesis, transport, and catabolism | basF |
| 387 | ZP_05828924.1 |  | Histidine decarboxylase | Amino acid transport and metabolism | basG |
| 388 | ZP_05828925.1 | 39 | ABC transporter | Energy production and conversion | CydD (barA) |
| 389 | ZP_05828926.1 |  | ABC transporter, CydDC cysteine exporter (CydDC-E) family, permease/ATP-binding protein CydC | Defense mechanisms | mdlB (barB) |
| 390 | ZP_05828927.1 |  | acinetobactin biosynthesis protein | Secondary metabolites biosynthesis, transport, and catabolism | GrsT (basH) |
| 391 | ZP_05828942.1 | 40 | TonB-dependent receptor | Inorganic ion transport and metabolism | FepA (tonB) |
| 392 | ZP_05828943.1 |  | aminopeptidase N | Amino acid transport and metabolism | PepN |
| 393 | ZP_05828978.1 |  | predicted protein |  |  |
| 394 | ZP_05828986.1 |  | integrase | DNA replication, recombination, and repair | XerD |
| 395 | ZP_05828987.1 |  | gp30 | Transcription | ND |
| 396 | ZP_05828988.1 |  | conserved hypothetical protein |  |  |
| 397 | ZP_05828989.1 |  | predicted protein |  |  |
| 398 | ZP_05828990.1 |  | conserved hypothetical protein |  |  |
| 399 | ZP_05828991.1 |  | conserved hypothetical protein |  |  |
| 400 | ZP_05828992.1 |  | conserved hypothetical protein |  |  |
| 401 | ZP_05828993.1 |  | exonuclease VIII |  |  |
| 402 | ZP_05828994.1 |  | conserved hypothetical protein |  |  |
| 403 | ZP_05828995.1 |  | conserved hypothetical protein |  |  |
| 404 | ZP_05828996.1 |  | predicted protein |  |  |
| 405 | ZP_05828997.1 |  | conserved hypothetical protein |  |  |
| 406 | ZP_05828998.1 |  | predicted protein | Function unknown | ND |
| 407 | ZP_05828999.1 |  | conserved hypothetical protein |  |  |
| 408 | ZP_05829000.1 |  | conserved hypothetical protein | Amino acid transport and metabolism | ND |
| 409 | ZP_05829001.1 |  | conserved hypothetical protein |  |  |
| 410 | ZP_05829002.1 |  | LexA repressor | Transcription | LexA |
| 411 | ZP_05829003.1 |  | predicted protein |  |  |
| 412 | ZP_05829005.1 |  | conserved hypothetical protein |  |  |
| 413 | ZP_05829006.1 |  | conserved hypothetical protein |  |  |
| 414 | ZP_05829007.1 | 41 | conserved hypothetical protein | General function prediction only | ND |
| 415 | ZP_05829008.1 |  | replicative DNA helicase | DNA replication, recombination, and repair | DnaB |
| 416 | ZP_05829009.1 |  | conserved hypothetical protein |  |  |
| 417 | ZP_05829010.1 |  | conserved hypothetical protein |  |  |
| 418 | ZP_05829011.1 |  | predicted protein |  |  |
| 419 | ZP_05829012.1 |  | predicted protein |  |  |
| 420 | ZP_05829013.1 |  | predicted protein |  |  |
| 421 | ZP_05829014.1 |  | conserved hypothetical protein |  |  |
| 422 | ZP_05829015.1 |  | PmgM |  |  |
| 423 | ZP_05829016.1 |  | predicted protein |  |  |
| 424 | ZP_05829017.1 |  | predicted protein |  |  |
| 425 | ZP_05829018.1 |  | conserved hypothetical protein |  |  |
| 426 | ZP_05829019.1 |  | predicted protein |  |  |
| 427 | ZP_05829020.1 |  | predicted protein |  |  |
| 428 | ZP_05829021.1 |  | predicted protein |  |  |
| 429 | ZP_05829022.1 |  | conserved hypothetical protein |  |  |
| 430 | ZP_05829023.1 | 42 | conserved hypothetical protein |  |  |
| 431 | ZP_05829024.1 |  | conserved hypothetical protein |  |  |
| 432 | ZP_05829027.1 |  | conserved hypothetical protein |  |  |
| 433 | ZP_05829028.1 |  | phage head morphogenesis protein | Function unknown | ND |
| 434 | ZP_05829029.1 | 43 | phage protein |  |  |
| 435 | ZP_05829030.1 |  | conserved hypothetical protein |  |  |
| 436 | ZP_05829031.1 |  | predicted protein |  |  |
| 437 | ZP_05829032.1 |  | conserved hypothetical protein |  |  |
| 438 | ZP_05829033.1 |  | glutamate 5-kinase |  |  |
| 439 | ZP_05829034.1 |  | predicted protein |  |  |
| 440 | ZP_05829035.1 |  | conserved hypothetical protein |  |  |
| 441 | ZP_05829036.1 |  | conserved hypothetical protein |  |  |
| 442 | ZP_05829037.1 |  | predicted protein |  |  |
| 443 | ZP_05829038.1 |  | predicted protein |  |  |
| 444 | ZP_05829039.1 |  | predicted protein |  |  |
| 445 | ZP_05829041.1 | 44 | phage integrase | DNA replication, recombination, and repair | XerD |
| 446 | ZP_05829042.1 |  | phage integrase |  |  |
| 447 | ZP_05829043.1 |  | DNA-binding protein | Function unknown | ND |
| 448 | ZP_05829044.1 |  | conserved hypothetical protein |  |  |
| 449 | ZP_05829045.1 |  | conserved hypothetical protein | DNA replication, recombination, and repair | ND |
| 450 | ZP_05829046.1 |  | predicted protein | Cell division and chromosome partitioning | StbD |
| 451 | ZP_05829047.1 |  | conserved hypothetical protein | Function unknown | ND |
| 452 | ZP_05829068.1 |  | conserved hypothetical protein |  |  |
| 453 | ZP_05829070.1 | 45 | conserved hypothetical protein |  |  |
| 454 | ZP_05829071.1 |  | RloA protein | General function prediction only | ND |
| 455 | ZP_05829075.1 |  | restriction endonuclease PvuRts1I |  |  |
| 456 | ZP_05829083.1 |  | predicted protein |  |  |
| 457 | ZP_05829145.1 | 46 | type 4 pilin | Cell motility and secretion | PilA |
| 458 | ZP_05829146.1 |  | conserved hypothetical protein |  |  |
| 459 | ZP_05829203.1 |  | conserved hypothetical protein | Function unknown | ND |
| 460 | ZP_05829287.1 |  | AraC family transcriptional regulator | Transcription | AraC |
| 461 | ZP_05829288.1 |  | DMT family permease | Function unknown | ND |
| 462 | ZP_05829330.1 |  | conserved hypothetical protein |  |  |
| 463 | ZP_05829336.1 | 47 | glycosyl transferase group 1 |  |  |
| 464 | ZP_05829338.1 |  | glycosyltransferase | Cell envelope biogenesis, outer membrane | WcaA |
| 465 | ZP_05829339.1 |  | glycosyltransferase | Cell envelope biogenesis, outer membrane | RfaG |
| 466 | ZP_05829340.1 |  | conserved hypothetical protein |  |  |
| 467 | ZP_05829342.1 |  | conserved hypothetical protein |  |  |
| 468 | ZP_05829344.1 |  | glycosyltransferase |  |  |
| 469 | ZP_05829352.1 |  | glutathione S-transferase | Posttranslational modification, protein turnover, chaperones | Gst |
| 470 | ZP_05829353.1 |  | LysR family transcriptional regulator | Transcription | LysR |
| 471 | ZP_05829391.1 |  | conserved hypothetical protein |  |  |
| 472 | ZP_05829490.1 |  | SAM-dependent methyltransferase | Cell envelope biogenesis, outer membrane | ND |
| 473 | ZP_05829528.1 |  | conserved hypothetical protein |  |  |
| 474 | ZP_05829541.1 |  | conserved hypothetical protein |  |  |
| 475 | ZP_05829542.1 |  | conserved hypothetical protein |  |  |
| 476 | ZP_05829543.1 |  | conserved hypothetical protein |  |  |
| 477 | ZP_05829544.1 |  | helix-turn-helix domain-containing protein | Transcription | ND |
| 478 | ZP_05829545.1 |  | conserved hypothetical protein |  |  |
| 479 | ZP_05829546.1 |  | conserved hypothetical protein |  |  |
| 480 | ZP_05829548.1 |  | conserved hypothetical protein |  |  |
| 481 | ZP_05829555.1 |  | outer membrane receptor protein | Inorganic ion transport and metabolism | CirA |
| 482 | ZP_05829557.1 |  | conserved hypothetical protein |  |  |
| 483 | ZP_05829558.1 |  | conserved hypothetical protein |  |  |
| 484 | ZP_05829591.1 |  | DNA integration/recombination/inversion protein | DNA replication, recombination, and repair | XerC |
| 485 | ZP_05829592.1 |  | predicted protein |  |  |
| 486 | ZP_05829593.1 |  | predicted protein | Transcription | AlpA |
| 487 | ZP_05829594.1 |  | predicted protein |  |  |
| 488 | ZP_05829595.1 |  | predicted protein |  |  |
| 489 | ZP_05829596.1 |  | predicted protein |  |  |
| 490 | ZP_05829597.1 |  | conserved hypothetical protein |  |  |
| 491 | ZP_05829598.1 |  | predicted protein |  |  |
| 492 | ZP_05829599.1 |  | predicted protein |  |  |
| 493 | ZP_05829600.1 |  | predicted protein |  |  |
| 494 | ZP_05829601.1 |  | predicted protein |  |  |
| 495 | ZP_05829602.1 |  | predicted protein |  |  |
| 496 | ZP_05829603.1 |  | conserved hypothetical protein |  |  |
| 497 | ZP_05829604.1 |  | GP47 | DNA replication, recombination, and repair | ND |
| 498 | ZP_05829605.1 |  | conserved hypothetical protein |  |  |
| 499 | ZP_05829606.1 |  | Cd(II)/Pb(II)-responsive transcriptional regulator | Transcription | SoxR |
| 500 | ZP_05829607.1 |  | heavy metal detoxification protein | Inorganic ion transport and metabolism | CzcD |
| 501 | ZP_05829608.1 |  | signal peptidase II | Cell envelope biogenesis, outer membrane | LspA |
| 502 | ZP_05829609.1 |  | transposase |  |  |
| 503 | ZP_05829610.1 |  | Hg(II)-responsive transcriptional regulator | Transcription | SoxR |
| 504 | ZP_05829611.1 |  | mercury resistance inner membrane protein |  |  |
| 505 | ZP_05829612.1 |  | mercuric reductase | Energy production and conversion | Lpd |
| 506 | ZP_05829613.1 |  | transposase TnpA | DNA replication, recombination, and repair | ND |
| 507 | ZP_05829614.1 |  | cell filamentation protein Fic | Cell division and chromosome partitioning | Fic |
| 508 | ZP_05829615.1 |  | predicted protein |  |  |
| 509 | ZP_05829617.1 | 48 | restriction endonuclease S subunit | Defense mechanisms | HsdS |
| 510 | ZP_05829618.1 |  | type I restriction enzyme | Defense mechanisms | ND |
| 511 | ZP_05829619.1 |  | GP47 | DNA replication, recombination, and repair | ND |
| 512 | ZP_05829620.1 |  | conserved hypothetical protein |  |  |
| 513 | ZP_05829621.1 |  | transcriptional regulator | Transcription | AcrR |
| 514 | ZP_05829622.1 |  | rhs element Vgr family protein | Function unknown | VgrG |
| 515 | ZP_05829623.1 | 49 | Zn-dependent protease with chaperone function | Posttranslational modification, protein turnover, chaperones | HtpX |
| 516 | ZP_05829624.1 |  | transmembrane protein | Function unknown | ND |
| 517 | ZP_05829627.1 |  | bacterial regulatory protein | Transcription | AcrR |
| 518 | ZP_05829628.1 |  | conserved hypothetical protein |  |  |
| 519 | ZP_05829629.1 | 50 | curli production assembly/transport component CsgG | Cell envelope biogenesis, outer membrane | CsgG |
| 520 | ZP_05829630.1 |  | lipoprotein | Function unknown | ND |
| 521 | ZP_05829631.1 |  | lipoprotein | Function unknown | ND |
| 522 | ZP_05829642.1 |  | arginine N-succinyltransferase | Amino acid transport and metabolism | AstA |
| 523 | ZP_05829644.1 |  | conserved hypothetical protein |  |  |
| 524 | ZP_05829645.1 |  | conserved hypothetical protein |  |  |
| 525 | ZP_05829646.1 |  | predicted protein |  |  |
| 526 | ZP_05829647.1 |  | predicted protein |  |  |
| 527 | ZP_05829651.1 | 51 | galactarate dehydratase | Carbohydrate transport and metabolism | UxaA |
| 528 | ZP_05829652.1 |  | D-glucarate/D-galactarate permease | Carbohydrate transport and metabolism | UhpC |
| 529 | ZP_05829653.1 |  | glucarate dehydratase | Cell envelope biogenesis, outer membrane | ND |
| 530 | ZP_05829654.1 | 52 | 5-dehydro-4-deoxyglucarate dehydratase | Amino acid transport and metabolism | DapA |
| 531 | ZP_05829655.1 |  | 2-hydroxymuconic semialdehyde dehydrogenase | Energy production and conversion | PutA |
| 532 | ZP_05829656.1 |  | transcriptional regulator | Transcription | FadR |
| 533 | ZP_05829659.1 |  | UPF0311 protein |  |  |
| 534 | ZP_05829683.1 |  | metal-dependent hydrolase | General function prediction only | ND |
| 535 | ZP_05829684.1 |  | 2-hydroxycyclohexanecarboxyl-CoA dehydrogenase | General function prediction only | DltE |
| 536 | ZP_05829685.1 |  | monooxygenase | Inorganic ion transport and metabolism | TrkA |
| 537 | ZP_05829694.1 |  | conserved hypothetical protein |  |  |
| 538 | ZP_05829695.1 |  | conserved hypothetical protein |  |  |
| 539 | ZP_05829696.1 |  | predicted protein |  |  |
| 540 | ZP_05829697.1 |  | conserved hypothetical protein |  |  |
| 541 | ZP_05829699.1 |  | predicted protein |  |  |
| 542 | ZP_05829701.1 |  | conserved hypothetical protein |  |  |
| 543 | ZP_05829704.1 |  | conserved hypothetical protein |  |  |
| 544 | ZP_05829705.1 |  | LexA repressor | Transcription | LexA |
| 545 | ZP_05829706.1 |  | conserved hypothetical protein | Function unknown | ND |
| 546 | ZP_05829707.1 |  | conserved hypothetical protein |  |  |
| 547 | ZP_05829709.1 |  | site-specific DNA-methyltransferase | DNA replication, recombination, and repair | Dcm |
| 548 | ZP_05829712.1 |  | conserved hypothetical protein |  |  |
| 549 | ZP_05829713.1 |  | predicted protein |  |  |
| 550 | ZP_05829714.1 |  | conserved hypothetical protein |  |  |
| 551 | ZP_05829715.1 |  | GP72 |  |  |
| 552 | ZP_05829716.1 |  | conserved hypothetical protein |  |  |
| 553 | ZP_05829717.1 |  | predicted protein |  |  |
| 554 | ZP_05829718.1 |  | predicted protein |  |  |
| 555 | ZP_05829719.1 |  | conserved hypothetical protein |  |  |
| 556 | ZP_05829720.1 | 53 | conserved hypothetical protein |  |  |
| 557 | ZP_05829722.1 |  | phage protein | Function unknown | ND |
| 558 | ZP_05829723.1 |  | large subunit terminase TerL | Function unknown | ND |
| 559 | ZP_05829724.1 |  | phage-associated protein | Function unknown | ND |
| 560 | ZP_05829725.1 |  | phage head morphogenesis protein | Function unknown | ND |
| 561 | ZP_05829726.1 |  | conserved hypothetical protein | Function unknown | ND |
| 562 | ZP_05829727.1 |  | conserved hypothetical protein |  |  |
| 563 | ZP_05829728.1 |  | predicted protein |  |  |
| 564 | ZP_05829729.1 |  | conserved hypothetical protein | Function unknown | ND |
| 565 | ZP_05829730.1 |  | conserved hypothetical protein |  |  |
| 566 | ZP_05829731.1 |  | conserved hypothetical protein | Function unknown | ND |
| 567 | ZP_05829732.1 |  | conserved hypothetical protein |  |  |
| 568 | ZP_05829733.1 |  | conserved hypothetical protein |  |  |
| 569 | ZP_05829734.1 |  | conserved hypothetical protein |  |  |
| 570 | ZP_05829735.1 |  | conserved hypothetical protein |  |  |
| 571 | ZP_05829736.1 |  | conserved hypothetical protein |  |  |
| 572 | ZP_05829737.1 |  | conserved hypothetical protein |  |  |
| 573 | ZP_05829738.1 |  | conserved hypothetical protein |  |  |
| 574 | ZP_05829739.1 |  | conserved hypothetical protein |  |  |
| 575 | ZP_05829740.1 | 54 | conserved hypothetical protein |  |  |
| 576 | ZP_05829741.1 |  | phage tail tape measure protein | Cell division and chromosome partitioning | Smc |
| 577 | ZP_05829742.1 |  | conserved hypothetical protein |  |  |
| 578 | ZP_05829743.1 |  | conserved hypothetical protein | Function unknown | ND |
| 579 | ZP_05829744.1 |  | cellulosome enzyme |  |  |
| 580 | ZP_05829745.1 |  | conserved hypothetical protein |  |  |
| 581 | ZP_05829746.1 |  | cell wall-associated hydrolase | Cell envelope biogenesis, outer membrane | Spr |
| 582 | ZP_05829747.1 |  | fibronectin type III domain-containing protein | Function unknown | ND |
| 583 | ZP_05829748.1 |  | conserved hypothetical protein |  |  |
| 584 | ZP_05829749.1 |  | peptidoglycan domain-containing protein | General function prediction only | zliS |
| 585 | ZP_05829751.1 |  | predicted protein |  |  |
| 586 | ZP_05829754.1 |  | phage integrase | DNA replication, recombination, and repair | XerC |
| 587 | ZP_05829755.1 |  | predicted protein |  |  |
| 588 | ZP_05829758.1 |  | conserved hypothetical protein |  |  |
| 589 | ZP_05829778.1 |  | conserved hypothetical protein |  |  |
| 590 | ZP_05829779.1 |  | predicted protein |  |  |
| 591 | ZP_05829780.1 |  | hemolysin-type calcium-binding region | Secondary metabolites biosynthesis, transport, and catabolism | ND |
| 592 | ZP_05829823.1 |  | streptomycin 3''-adenylyltransferase |  |  |
| 593 | ZP_05829867.1 |  | conserved hypothetical protein |  |  |
| 594 | ZP_05829870.1 |  | 3-oxoacyl-[acyl-carrier-protein] synthase 2 | Lipid metabolism | FabB |
| 595 | ZP_05829872.1 |  | conserved hypothetical protein |  |  |
| 596 | ZP_05829873.1 |  | conserved hypothetical protein |  |  |
| 597 | ZP_05829876.1 |  | conserved hypothetical protein |  |  |
| 598 | ZP_05829877.1 |  | conserved hypothetical protein |  |  |
| 599 | ZP_05829878.1 |  | conserved hypothetical protein |  |  |
| 600 | ZP_05829896.1 | 55 | conserved hypothetical protein |  |  |
| 601 | ZP_05829897.1 |  | conserved hypothetical protein | Cell envelope biogenesis, outer membrane | RfaG |
| 602 | ZP_05829898.1 |  | glycosyltransferase | Cell envelope biogenesis, outer membrane | RfaG |
| 603 | ZP_05829899.1 |  | capsular polysaccharide synthesis enzyme | General function prediction only | WbbJ |
| 604 | ZP_05829900.1 | 56 | O-antigen translocase | General function prediction only | RfbX |
| 605 | ZP_05829901.1 |  | UDP-4-keto-6-deoxy-N-acetylglucosamine 4-aminotransferase | Cell envelope biogenesis, outer membrane | WecE |
| 606 | ZP_05829902.1 |  | WbbJ protein | Amino acid transport and metabolism | CysE |
| 607 | ZP_05829903.1 |  | MviM protein | General function prediction only | MviM |
| 608 | ZP_05829915.1 |  | predicted protein |  |  |
| 609 | ZP_05829916.1 |  | predicted protein |  |  |
| 610 | ZP_05829917.1 |  | predicted protein |  |  |
| 611 | ZP_05829918.1 |  | predicted protein |  |  |
| 612 | ZP_05829919.1 |  | predicted protein |  |  |
| 613 | ZP_05829920.1 |  | predicted protein |  |  |
| 614 | ZP_05829921.1 |  | predicted protein |  |  |
| 615 | ZP_05829922.1 |  | predicted protein |  |  |
| 616 | ZP_05829923.1 |  | conserved hypothetical protein |  |  |
| 617 | ZP_05829924.1 |  | predicted protein |  |  |
| 618 | ZP_05829925.1 |  | predicted protein |  |  |
| 619 | ZP_05829926.1 |  | predicted protein |  |  |
| 620 | ZP_05829927.1 |  | predicted protein |  |  |
| 621 | ZP_05829928.1 |  | predicted protein |  |  |
| 622 | ZP_05829929.1 |  | predicted protein |  |  |
| 623 | ZP_05829930.1 |  | conserved hypothetical protein |  |  |
| 624 | ZP_05829931.1 |  | resolvase domain-containing protein | DNA replication, recombination, and repair | PinR |
| 625 | ZP_05829932.1 |  | fic protein family | Cell division and chromosome partitioning | Fic |
| 626 | ZP_05829933.1 |  | predicted protein |  |  |
| 627 | ZP_05829934.1 |  | conserved hypothetical protein |  |  |
| 628 | ZP_05829935.1 |  | conserved hypothetical protein |  |  |
| 629 | ZP_05829936.1 |  | dihydropteroate synthase | Coenzyme metabolism | FolP |
| 630 | ZP_05829937.1 |  | phosphoglucosamine mutase | Carbohydrate transport and metabolism | ManB |
| 631 | ZP_05829938.1 |  | transposase iscr2 |  |  |
| 632 | ZP_05829939.1 |  | conserved hypothetical protein |  |  |
| 633 | ZP_05829940.1 |  | resolvase | DNA replication, recombination, and repair | PinR |
| 634 | ZP_05829941.1 |  | transcriptional regulator | Transcription | ArsR |
| 635 | ZP_05829943.1 |  | arsenical-resistance protein | Inorganic ion transport and metabolism | ACR3 |
| 636 | ZP_05829944.1 |  | conserved hypothetical protein |  |  |
| 637 | ZP_05829945.1 |  | conserved hypothetical protein |  |  |
| 638 | ZP_05829946.1 |  | conserved hypothetical protein |  |  |
| 639 | ZP_05829947.1 |  | P-type conjugative transfer protein TrbL | Intracellular trafficking and secretion | TrbL |
| 640 | ZP_05829948.1 |  | conserved hypothetical protein |  |  |
| 641 | ZP_05829949.1 |  | P-type conjugative transfer protein TrbJ | Intracellular trafficking and secretion | ND |
| 642 | ZP_05829950.1 |  | conserved hypothetical protein | General function prediction only | StbC |
| 643 | ZP_05829951.1 |  | replication C family protein |  |  |
| 644 | ZP_05829952.1 |  | replication protein | DNA replication, recombination, and repair | RepA |
| 645 | ZP_05829953.1 |  | phage transcriptional regulator | Transcription | AlpA |
| 646 | ZP_05829954.1 |  | phage integrase | DNA replication, recombination, and repair | XerC |
| 647 | ZP_05829955.1 | 57 | conserved hypothetical protein |  |  |
| 648 | ZP_05829956.1 |  | predicted protein |  |  |
| 649 | ZP_05829957.1 |  | predicted protein |  |  |
| 650 | ZP_05829958.1 |  | conserved hypothetical protein |  |  |
| 651 | ZP_05829959.1 |  | predicted protein |  |  |
| 652 | ZP_05829960.1 |  | conserved hypothetical protein |  |  |
| 653 | ZP_05829961.1 |  | predicted protein |  |  |
| 654 | ZP_05829962.1 |  | predicted protein |  |  |
| 655 | ZP_05829984.1 |  | isoleucyl-tRNA synthetase | Translation, ribosomal structure and biogenesis | IleS |
| 656 | ZP_05830014.1 |  | conserved hypothetical protein |  |  |
| 657 | ZP_05830043.1 |  | conserved hypothetical protein |  |  |
| 658 | ZP_05830054.1 |  | phosphatase | General function prediction only | Gph |
| 659 | ZP_05830068.1 |  | catalase | Inorganic ion transport and metabolism | KatE |
| 660 | ZP_05830069.1 |  | ankyrin repeat-containing protein | General function prediction only | Arp |
| 661 | ZP_05830086.1 | 58 | glycoside hydrolase, family 19 | General function prediction only | ND |
| 662 | ZP_05830087.1 |  | predicted protein |  |  |
| 663 | ZP_05830091.1 |  | 2-hydroxycyclohexanecarboxyl-CoA dehydrogenase | General function prediction only | DltE |
| 664 | ZP_05830128.1 |  | Fe-S-cluster oxidoreductase | General function prediction only | ND |
| 665 | ZP_05830135.1 |  | conserved hypothetical protein |  |  |
| 666 | ZP_05830165.1 |  | outer membrane protein | Cell envelope biogenesis, outer membrane | TolC |
| 667 | ZP_05830172.1 |  | shufflon-specific DNA recombinase | DNA replication, recombination, and repair | XerD |
| 668 | ZP_05830173.1 |  | conserved hypothetical protein |  |  |
| 669 | ZP_05830174.1 | 59 | peptidoglycan domain-containing protein | General function prediction only | zliS |
| 670 | ZP_05830175.1 |  | conserved hypothetical protein |  |  |
| 671 | ZP_05830176.1 |  | conserved hypothetical protein |  |  |
| 672 | ZP_05830177.1 |  | cellulosome enzyme |  |  |
| 673 | ZP_05830178.1 |  | predicted protein |  |  |
| 674 | ZP_05830179.1 |  | predicted protein |  |  |
| 675 | ZP_05830180.1 |  | predicted protein |  |  |
| 676 | ZP_05830181.1 | 60 | predicted protein |  |  |
| 677 | ZP_05830182.1 |  | predicted protein |  |  |
| 678 | ZP_05830183.1 |  | conserved hypothetical protein |  |  |
| 679 | ZP_05830184.1 |  | Bbp13 |  |  |
| 680 | ZP_05830185.1 |  | predicted protein |  |  |
| 681 | ZP_05830186.1 |  | predicted protein |  |  |
| 682 | ZP_05830187.1 |  | predicted protein |  |  |
| 683 | ZP_05830188.1 | 61 | Bbp17 |  |  |
| 684 | ZP_05830189.1 |  | predicted protein |  |  |
| 685 | ZP_05830190.1 |  | predicted protein |  |  |
| 686 | ZP_05830191.1 |  | Bbp21 |  |  |
| 687 | ZP_05830192.1 |  | predicted protein |  |  |
| 688 | ZP_05830193.1 |  | phage terminase large subunit |  |  |
| 689 | ZP_05830194.1 |  | hypothetical protein | DNA replication, recombination, and repair | XtmA |
| 690 | ZP_05830195.1 |  | predicted protein |  |  |
| 691 | ZP_05830196.1 |  | predicted protein |  |  |
| 692 | ZP_05830197.1 | 62 | DNA replication protein | DNA replication, recombination, and repair | DnaC |
| 693 | ZP_05830198.1 |  | predicted protein | Function unknown | ND |
| 694 | ZP_05830199.1 |  | D12 class N6 adenine-specific DNA methyltransferase | DNA replication, recombination, and repair | Dam |
| 695 | ZP_05830200.1 |  | predicted protein |  |  |
| 696 | ZP_05830201.1 |  | Upf86.8 |  |  |
| 697 | ZP_05830202.1 |  | predicted protein |  |  |
| 698 | ZP_05830203.1 | 63 | predicted protein |  |  |
| 699 | ZP_05830204.1 |  | conserved hypothetical protein |  |  |
| 700 | ZP_05830205.1 |  | predicted protein |  |  |
| 701 | ZP_05830206.1 |  | transcriptional regulator | Transcription | ND |
| 702 | ZP_05830207.1 |  | predicted protein |  |  |
| 703 | ZP_05830208.1 | 64 | conserved hypothetical protein | Function unknown | ND |
| 704 | ZP_05830209.1 |  | predicted protein |  |  |
| 705 | ZP_05830210.1 |  | YqaJ | DNA replication, recombination, and repair | ND |
| 706 | ZP_05830211.1 |  | transcriptional regulator |  |  |
| 707 | ZP_05830212.1 |  | superfamily I DNA and RNA helicase | DNA replication, recombination, and repair | UvrD |
| 708 | ZP_05830213.1 |  | predicted protein | Transcription | Fis |
| 709 | ZP_05830214.1 |  | conserved hypothetical protein |  |  |
| 710 | ZP_05830215.1 |  | predicted protein |  |  |
| 711 | ZP_05830216.1 |  | predicted protein |  |  |
| 712 | ZP_05830217.1 |  | conserved hypothetical protein |  |  |
| 713 | ZP_05830218.1 |  | predicted protein |  |  |
| 714 | ZP_05830219.1 |  | predicted protein | Function unknown | ND |
| 715 | ZP_05830220.1 |  | predicted protein |  |  |
| 716 | ZP_05830221.1 |  | conserved hypothetical protein |  |  |
| 717 | ZP_05830222.1 |  | conserved hypothetical protein |  |  |
| 718 | ZP_05830223.1 |  | predicted protein |  |  |
| 719 | ZP_05830224.1 |  | surface antigen | Cell envelope biogenesis, outer membrane | SlyB |
| 720 | ZP_05830225.1 |  | conserved hypothetical protein | Function unknown | ND |
| 721 | ZP_05830226.1 |  | peptidoglycan domain-containing protein |  |  |
| 722 | ZP_05830230.1 |  | phage head morphogenesis protein |  |  |
| 723 | ZP_05830231.1 |  | conserved hypothetical protein |  |  |
| 724 | ZP_05830232.1 |  | conserved hypothetical protein |  |  |
| 725 | ZP_05830233.1 |  | conserved hypothetical protein |  |  |
| 726 | ZP_05830234.1 |  | conserved hypothetical protein |  |  |
| 727 | ZP_05830240.1 |  | conserved hypothetical protein |  |  |
| 728 | ZP_05830268.1 |  | predicted protein |  |  |
| 729 | ZP_05830283.1 |  | conserved hypothetical protein |  |  |
| 730 | ZP_05830309.1 |  | dedA | Function unknown | DedA |
| 731 | ZP_05830318.1 |  | predicted protein |  |  |
| 732 | ZP_05830343.1 |  | conserved hypothetical protein |  |  |
| 733 | ZP_05830348.1 |  | conserved hypothetical protein |  |  |
| 734 | ZP_05830349.1 | 65 | DNA replication protein |  |  |
| 735 | ZP_05830350.1 |  | replicase | DNA replication, recombination, and repair | ND |
| 736 | ZP_05830351.1 |  | conserved hypothetical protein |  |  |
| 737 | ZP_05830352.1 |  | conserved hypothetical protein |  |  |
| 738 | ZP_05830353.1 |  | conserved hypothetical protein |  |  |
| 739 | ZP_05830354.1 |  | conserved hypothetical protein |  |  |
| 740 | ZP_05830355.1 |  | predicted protein |  |  |
| 741 | ZP_05830356.1 |  | predicted protein |  |  |
| 742 | ZP_05830360.1 |  | helix-turn-helix domain-containing protein | Function unknown | ND |
| 743 | ZP_05830361.1 |  | hth | Transcription | ND |
| 744 | ZP_05830362.1 |  | conserved hypothetical protein |  |  |
| 745 | ZP_05830363.1 | 66 | DNA replication protein |  |  |
| 746 | ZP_05830364.1 |  | DNA replication protein | DNA replication, recombination, and repair | ND |
| 747 | ZP_05830365.1 |  | conserved hypothetical protein |  |  |
| 748 | ZP_05830366.1 |  | plasmid mobilization protein |  |  |
| 749 | ZP_05830367.1 |  | predicted protein |  |  |
| 750 | ZP_05830368.1 | 67 | diaminopimelate decarboxylase | Function unknown | ND |
| 751 | ZP_05830369.1 |  | CRO family transcriptional regulator | Transcription | ND |
| 752 | ZP_05830370.1 |  | conserved hypothetical protein |  |  |
| 753 | ZP_05830371.1 | 68 | TPR repeat-containing SEL1 subfamily protein | General function prediction only | ND |
| 754 | ZP_05830372.1 |  | conserved hypothetical protein |  |  |
| 755 | ZP_05830373.1 | 69 | organic hydroperoxide resistance protein | Secondary metabolites biosynthesis, transport and catabolism | osmC |
| 756 | ZP_05830374.1 |  | organic hydroperoxide resistance transcriptional regulator | Transcription | MarR |
| 757 | ZP_05830375.1 |  | conserved hypothetical protein |  |  |
| 758 | ZP_05830376.1 |  | conserved hypothetical protein |  |  |
| 759 | ZP_05830381.1 |  | transposase (IS4 family) |  |  |
