# Supplementary material for: The Success of Acinetobacter Species; Genetic, Metabolic and Virulence Attributes
Source: PLoS One. 2012 Oct 29;7(10):e46984. doi: 10.1371/journal.pone.0046984 (PMC3483291; doi:10.1371/journal.pone.0046984)
Supplement: Table S3 — Metabolic gains found in A. baumannii ATCC 19606T but not in A. calcoaceticus using phenotypic microarrays (PM). (DOC) [file pone.0046984.s004.doc]

| **Supplementary Table S3 -** Metabolic capabilities found in *A. baumannii* ATCC 19606^T^ but not in *A. calcoaceticus* using phenotypic microarrays (PM). | | | |
| --- | --- | --- | --- |
| **Plate** | **Well** | **Compound name** | **Compound type** |
| PM01 | A04 | D-Saccharic Acid | C-source |
| PM01 | F08 | Mucic Acid | C-source |
| PM01 | G02 | Tricarballylic Acid | C-source |
| PM01 | H07 | Glucuronamide | C-source |
| PM02 | E03 | Citraconic Acid | C-source |
| PM02 | E04 | D,L-Citramalic Acid | C-source |
| PM02 | F02 | Malonic Acid | C-source |
| PM02 | G08 | 4-Hydroxy-L-Proline (trans) | C-source |
| PM02 | H08 | Putrescine | C-source |
| PM02 | H10 | 2,3-Butanediol | C-source |
| PM03B | A03 | Nitrite | N-source |
| PM03B | A04 | Nitrate | N-source |
| PM03B | B11 | L-Threonine | N-source |
| PM03B | B12 | L-Tryptophan | N-source |
| PM03B | C01 | L-Tyrosine | N-source |
| PM03B | D11 | Putrescine | N-source |
| PM03B | F02 | Adenine | N-source |
| PM03B | H03 | Ala-Glu | N-source |
| PM03B | H07 | Ala-Thr | N-source |
| PM03B | H09 | Gly-Gln | N-source |
| PM06 | A06 | Ala-Glu | N-source |
| PM06 | A07 | Ala-Gly | N-source |
| PM06 | A10 | Ala-Lys | N-source |
| PM06 | A12 | Ala-Pro | N-source |
| PM06 | B07 | Arg-Asp | N-source |
| PM06 | C07 | Asn-Glu | N-source |
| PM06 | C09 | Asp-Asp | N-source |
| PM06 | C12 | Asp-Lys | N-source |
| PM06 | D02 | Asp-Trp | N-source |
| PM06 | D03 | Asp-Val | N-source |
| PM06 | D10 | Glu-Ser | N-source |
| PM06 | D12 | Glu-Tyr | N-source |
| PM06 | E01 | Glu-Val | N-source |
| PM06 | E11 | Gly-Pro | N-source |
| PM06 | E12 | Gly-Ser | N-source |
| PM06 | F04 | Gly-Val | N-source |
| PM06 | F12 | His-Trp | N-source |
| PM06 | G01 | His-Tyr | N-source |
| PM06 | G03 | Ile-Ala | N-source |
| PM06 | H09 | Leu-Ile | N-source |
| PM06 | H10 | Leu-Leu | N-source |
| PM06 | H11 | Leu-Met | N-source |
| PM06 | H12 | Leu-Phe | N-source |
| PM07 | A04 | Leu-Trp | N-source |
| PM07 | A05 | Leu-Val | N-source |
| PM07 | A06 | Lys-Ala | N-source |
| PM07 | A09 | Lys-Ile | N-source |
| PM07 | A10 | Lys-Leu | N-source |
| PM07 | B01 | Lys-Pro | N-source |
| PM07 | B04 | Lys-Trp | N-source |
| PM07 | B05 | Lys-Tyr | N-source |
| PM07 | B06 | Lys-Val | N-source |
| PM07 | B08 | Met-Asp | N-source |
| PM07 | B10 | Met-Glu | N-source |
| PM07 | C01 | Met-Ile | N-source |
| PM07 | C02 | Met-Leu | N-source |
| PM07 | C04 | Met-Met | N-source |
| PM07 | C05 | Met-Phe | N-source |
| PM07 | C06 | Met-Pro | N-source |
| PM07 | C07 | Met-Trp | N-source |
| PM07 | C11 | Phe-Ile | N-source |
| PM07 | C12 | Phe-Phe | N-source |
| PM07 | D01 | Phe-Pro | N-source |
| PM07 | D03 | Phe-Trp | N-source |
| PM07 | D04 | Pro-Ala | N-source |
| PM07 | D06 | Pro-Gln | N-source |
| PM07 | D07 | Pro-Gly | N-source |
| PM07 | D09 | Pro-Leu | N-source |
| PM07 | D10 | Pro-Phe | N-source |
| PM07 | D11 | Pro-Pro | N-source |
| PM07 | E01 | Ser-Ala | N-source |
| PM07 | E02 | Ser-Gly | N-source |
| PM07 | E05 | Ser-Met | N-source |
| PM07 | E07 | Ser-Pro | N-source |
| PM07 | F05 | Thr-Pro | N-source |
| PM07 | F08 | Trp-Asp | N-source |
| PM07 | F09 | Trp-Glu | N-source |
| PM07 | F11 | Trp-Leu | N-source |
| PM07 | G01 | Trp-Phe | N-source |
| PM07 | G03 | Trp-Trp | N-source |
| PM07 | G04 | Trp-Tyr | N-source |
| PM07 | G07 | Tyr-Glu | N-source |
| PM07 | G08 | Tyr-Gly | N-source |
| PM07 | G11 | Tyr-Lys | N-source |
| PM07 | G12 | Tyr-Phe | N-source |
| PM07 | H01 | Tyr-Trp | N-source |
| PM07 | H02 | Tyr-Tyr | N-source |
| PM07 | H06 | Val-Gly | N-source |
| PM07 | H08 | Val-Ile | N-source |
| PM07 | H09 | Val-Leu | N-source |
| PM07 | H10 | Val-Tyr | N-source |
| PM07 | H11 | Val-Val | N-source |
| PM08 | A03 | Ala-Asp | N-source |
| PM08 | A10 | Asp-Gly | N-source |
| PM08 | A11 | Glu-Ala | N-source |
| PM08 | B07 | Ile-Leu | N-source |
| PM08 | B10 | Leu-Pro | N-source |
| PM08 | B11 | Leu-Tyr | N-source |
| PM08 | C05 | Phe-Asp | N-source |
| PM08 | C06 | Phe-Glu | N-source |
| PM08 | C09 | Phe-Tyr | N-source |
| PM08 | C11 | Pro-Arg | N-source |
| PM08 | D04 | Pro-Ser | N-source |
| PM08 | D12 | Thr-Gln | N-source |
| PM08 | E01 | Thr-Phe | N-source |
| PM08 | E04 | Tyr-lle | N-source |
| PM08 | E05 | Tyr-Val | N-source |
| PM08 | E10 | Val-Met | N-source |
| PM08 | E11 | Val-Phe | N-source |
| PM08 | E12 | Val-Pro | N-source |
| PM08 | F01 | Val-Ser | N-source |
| PM08 | G01 | g-Glu-Gly | N-source |
| PM08 | G08 | Leu-b-Ala | N-source |
| PM08 | G10 | Phe-b-Ala | N-source |
| PM08 | H10 | Leu-Leu-Leu | N-source |
| PM09 | E01 | 1% Sodium Formate | osmotic sensitivity, sodium formate |
| PM09 | E02 | 2% Sodium Formate | osmotic sensitivity, sodium formate |
| PM09 | G05 | 20mM Sodium Benzoate pH 5.2 | toxicity, benzoate |
| PM09 | H08 | 20mM Sodium Nitrite | toxicity, nitrite |
| PM10 | B03 | pH 4.5 + L-Arginine | pH, decarboxylase |
| PM10 | B05 | pH 4.5 + L-Aspartic Acid | pH, decarboxylase |
| PM10 | B06 | pH 4.5 + L-Glutamic Acid | pH, decarboxylase |
| PM10 | B07 | pH 4.5 + L-Glutamine | pH, decarboxylase |
| PM10 | B08 | pH 4.5 + Glycine | pH, decarboxylase |
| PM10 | B09 | pH 4.5 + L-Histidine | pH, decarboxylase |
| PM10 | C07 | pH 4.5 + L-Tyrosine | pH, decarboxylase |
| PM10 | C08 | pH 4.5 + L-Valine | pH, decarboxylase |
| PM10 | C09 | pH 4.5 + Hydroxy-L-Proline | pH, decarboxylase |
| PM10 | C10 | pH 4.5 + L-Ornithine | pH, decarboxylase |
| PM10 | C11 | pH 4.5 + L-Homoarginine | pH, decarboxylase |
| PM10 | D03 | pH 4.5 + L-Norvaline | pH, decarboxylase |
| PM10 | D04 | pH 4.5 + a- Amino-N-Butyric Acid | pH, decarboxylase |
| PM10 | D06 | pH 4.5 + b-Hydroxy Glutamate | pH, decarboxylase |
| PM10 | D07 | pH 4.5 + g-Hydroxy Glutamic Acid | pH, decarboxylase |
| PM10 | D08 | pH 4.5 + 5-Hydroxy-L-Lysine | pH, decarboxylase |
| PM10 | E04 | pH 9.5 + L-Asparagine | pH, deaminase |
| PM10 | E05 | pH 9.5 + L-Aspartic Acid | pH, deaminase |
| PM10 | F05 | pH 9.5 + L-Threonine | pH, deaminase |
| PM10 | F08 | pH 9.5 + L-Valine | pH, deaminase |
| PM10 | F10 | pH 9.5 + L-Ornithine | pH, deaminase |
| PM10 | G04 | pH 9.5 + Agmatine | pH, deaminase |
| PM11C | A05 | Chlortetracycline | protein synthesis; 30S ribosomal subunit; tetracycline |
| PM11C | B04 | Amoxicillin | wall; lactam |
| PM11C | C07 | Colistin | membrane; transport |
| PM11C | E10 | Nalidixic acid | DNA unwinding; gyrase (GN); topoisomerase (GP); quinolone |
| PM12B | A03 | Penicillin G | wall, lactam |
| PM12B | A04 | Penicillin G | wall, lactam |
| PM12B | B06 | Penimepicycline | protein synthesis, tetracycline |
| PM12B | B12 | Polymyxin B | membrane, outer |
| PM12B | H06 | Rifampicin | RNA polymerase |
| PM12B | H11 | Dodecyltrimethyl Ammonium Bromide | membrane, detergent, cationic |
| PM13B | A12 | Nickel chloride | toxic cation |
| PM13B | B11 | Oxolinic acid | DNA unwinding, gyrase (GN), topoisomerase (GP), quinolone |
| PM13B | D11 | Rolitetracycline | protein synthesis, 30S ribosomal subunit, tetracycline |
| PM14 | B07 | Fusaric Acid | chelator, lipophilic |
| PM14 | H07 | Promethazine | cyclic nucleotide phosphodiesterase |
| PM15B | A07 | Guanidine hydrochloride | membrane, chaotropic agent |
| PM15B | B07 | EDTA | chelator, hydrophilic |
| PM15B | C05 | Fusidic acid | protein synthesis, elongation factor |
| PM15B | D07 | Domiphen bromide | membrane, detergent, cationic, fungiside |
| PM15B | E03 | Alexidine | membrane, biguanide, electron transport |
| PM15B | G10 | Menadione | respiration, uncoupler |
| PM16A | C01 | Dichlofluanid | fungicide, phenylsulphamide |
| PM16A | C02 | Dichlofluanid | fungicide, phenylsulphamide |
| PM16A | C03 | Dichlofluanid | fungicide, phenylsulphamide |
| PM16A | C12 | Cetylpyridinium Chloride | membrane, detergent, cationic |
| PM16A | D12 | Cinoxacin | protein synthesis |
| PM16A | F02 | Potassium Tellurite | transport, toxic anion |
| PM17A | A08 | b-Chloro-L-Alanine | aa analog, alanine, aminotransferase inhibitor |
| PM17A | B02 | Salicylate | anti-capsule, anti-inflammatory, mar inducer |
| PM17A | E04 | Niaproof | membrane, detergent, anionic |
| PM17A | E08 | Compound 48/80 | phospholipase C, ADP ribosylation |
| PM17A | E11 | Sodium Tungstate | transport, toxic anion, molybdate analog |
| PM18C | C11 | Pentachlorophenol (PCP) | respiration, ionophore, H+ |
| PM18C | H06 | 2- Phenylphenol | DNA intercalator |
| PM18C | H10 | Plumbagin | oxidizing agent |
| PM19 | B04 | Methyltrioctylammonium Chloride | membrane, detergent, cationic |
| PM19 | B07 | Harmane | imidazoline binding sites, agonist |
| PM19 | C06 | Umbelliferone | DNA intercalator |
| PM19 | D02 | Disulphiram | fungicide |
| PM19 | D12 | Phenyl-Methyl-Sulfonyl-Fluoride (PMSF) | protease inhibitor, serine |
| PM19 | E07 | D,L-Thioctic Acid | oxidizing agent |
| PM20B | A03 | Amitriptyline | membrane, transport |
| PM20B | C12 | Ornidazole | protein glycosolation |
| PM20B | E06 | Dodine | fungicide, guanidine, membrane permeability |
| PM20B | F06 | Oxytetracycline | protein synthesis, tetracycline |
| PM20B | F07 | Oxytetracycline | protein synthesis, tetracycline |
| PM20B | F11 | Pridinol | cholinergic antagonist |
| PM20B | G01 | Captan | fungicide, carbamate, multisite |
| PM20B | G02 | Captan | fungicide, carbamate, multisite |
| PM20B | G04 | Captan | fungicide, carbamate, multisite |
| PM20B | H05 | Tolylfluanid | fungicide, phenylsulphamide |
| PM20B | H06 | Tolylfluanid | fungicide, phenylsulphamide |
| PM20B | H07 | Tolylfluanid | fungicide, phenylsulphamide |
| PM20B | H08 | Tolylfluanid | fungicide, phenylsulphamide |
