# Supplementary material for: The Success of Acinetobacter Species; Genetic, Metabolic and Virulence Attributes
Source: PLoS One. 2012 Oct 29;7(10):e46984. doi: 10.1371/journal.pone.0046984 (PMC3483291; doi:10.1371/journal.pone.0046984)
Supplement: Text S1 — Materials and Methods. (DOC) [file pone.0046984.s005.doc]

**SUPPLEMENTARY MATERIAL**

**Methods**

**Whole genome sequencing**

Genomes were assembled using Newbler and the runAssembly script was then used to assemble reads into contigs. Final assemblies were BLASTed to the NCBI non-redundant database and UniVecCore was used to remove any contaminating sequence. For annotation, *ab initio* gene models were predicted using GeneMark, Glimmer3, MetaGene, and Zcurveb . An evidence-based approach constructed open reading frames (ORFs) from BLASTX hits with the NCBI non–redundant protein database; all BLAST hits with e–values better than 1e–10 were used as BLAST evidence. A summary of gene finding data for each locus can be viewed at the Broad Institute *Acinetobacter* group database (http://www.broadinstitute.org/annotation/genome/Acinetobacter_group/GenomeStats.html).

**Metabolic Profiling**

Twenty 96–well microarray plates were used (PM 1-20) comprising 1920 different metabolic and toxic compound conditions, including 192 assays of C-source metabolism (PM 1–2), 384 assays of N–source metabolism (PM 3, 6 – 8), 96 assays of P-source and S–source metabolism (PM 4), 96 assays of biosynthetic pathways (PM 5), 96 assays of ion effects and osmolarity (PM 9), 96 assays of pH effects (PM 10), and sensitivity to 240 chemicals (PM 11–20) . In brief, a standardized bacterial cell density suspension containing a tetrazolium redox dye (measures cell respiration) was transferred into wells of the microplates, which contained dried nutrients or chemicals to create the unique culture conditions. Plates were incubated at 37°C and metabolic activity was measured colorimetrically using the redox dye . Bioinformatic software (OmniLog V. 1.5) quantified metabolism as a color–coded kinetic graph. Two independent experiments were performed for all analyses. A best blast hit approach was used to map all the proteins in the four genomes to the KEGG reactions database using the KEGG proteome database (<http://www.genome.jp/kegg/>, release 54.1); an E–value threshold of 1^e–50^ was applied. Phenotype microarray data was analyzed using GenoPhenomicon: the activity of each well was predicted as *active/not active* using a support vector machine (SVM) predictor built using SVMpython v.2 (http://www.tfinley.net/software/svmpython2/), using a training dataset of 800 manually validated samples. Relationships between genome content and phenotype microarray data were then inspected.

**References**

1. Delcher AL, Harmon D, Kasif S, White O, Salzberg SL (1999) Improved microbial gene identification with GLIMMER. Nucleic Acids Res 27: 4636-4641.

2. Guo FB, Ou HY, Zhang CT (2003) ZCURVE: a new system for recognizing protein-coding genes in bacterial and archaeal genomes. Nucleic Acids Res 31: 1780-1789.

3. Noguchi H, Park J, Takagi T (2006) MetaGene: prokaryotic gene finding from environmental genome shotgun sequences. Nucleic Acids Res 34: 5623-5630.

4. Bochner BR (2009) Global phenotypic characterization of bacteria. FEMS Microbiol Rev 33: 191-205.
